# Photochemical and electrochemical assessment of UIO-66-NH₂/g-C₃N₄ thin-film heterostructures as potential candidates for hydrogen evolution: an experimental study augmented by DFT insights

**DOI:** 10.1038/s41598-025-20035-4

**Published:** 2025-09-18

**Authors:** Nour AbouSeada, Maryam G. Elmahgary, Sameh O. Abdellatif, Khaled Kirah

**Affiliations:** 1 The Faculty of Energy and Environmental Engineering, The British University in Egypt (BUE), El-Sherouk City, Cairo 11837 Egypt; 2 The Chemical Engineering department, British University in Egypt (BUE), El-Sherouk City, Cairo 11387 Egypt; 3 The Electrical Engineering department, and FabLab, Centre of Emerging Learning Technologies, CELT, British University in Egypt (BUE), El-Sherouk City, Cairo 11387 Egypt; 4 Engineering Physics Department, Faculty of Engineering, Ain Shams University, Cairo, Egypt

**Keywords:** Zirconium-Based Metal-Organic frameworks, Graphitic carbon nitride, Sustainable energy, Photocatalysis, Electrocatalysis, Energy conversion, Heterojunctions, Environmental sustainability, Chemistry, Energy science and technology, Materials science, Nanoscience and technology

## Abstract

The global shift towards carbon-neutral energy systems has catalyzed an intensified focus on sustainable hydrogen production, with photo and electrochemical water splitting emerging as a particularly promising pathway. This study elucidates the design, simulation, and synthesis of advanced photo and electrocatalytic materials tailored for the hydrogen evolution reaction (HER), concentrating on heterostructures formed by zirconium-based metal-organic frameworks (MOFs)—specifically, UiO-66 and its amine-functionalized derivative, UiO-66-NH₂—in conjunction with graphitic carbon nitride (g-C₃N₄). Employing density functional theory (DFT) simulations, we pre-screened the electronic properties of the MOFs, revealing that amine functionalization significantly narrows the bandgap and optimizes band alignment, thereby enhancing photocatalytic activity. Guided by DFT-derived analyses of electronic structure and density of states, UiO-66-NH₂ was selected for experimental synthesis. Thin-film catalysts comprising UiO-66-NH₂ and g-C₃N₄ in varying weight ratios (60:40, 70:30, and 50:50) were deposited onto fluorine-doped tin oxide (FTO) substrates and subsequently evaluated in a standard three-electrode photochemical setup using a 0.5 M Na₂SO₃ electrolyte, followed by testing in an electrochemical configuration with 1 M KOH. Comprehensive material characterization techniques—including X-ray diffraction (XRD), scanning and transmission electron microscopy (SEM/TEM), energy-dispersive X-ray spectroscopy (EDX), X-ray photoelectron spectroscopy (XPS), and ultraviolet-visible diffuse reflectance spectroscopy (UV-Vis DRS)—coupled with rigorous electrochemical assessments (linear sweep voltammetry (LSV), cyclic voltammetry (CV), chronoamperometry (CA), and electrochemical impedance spectroscopy (EIS)), demonstrated that the 70:30 UiO-66-NH₂/g-C₃N₄ composite exhibited superior HER performance. This composite achieved the highest stable photocurrent response, a low overpotential of 135 mV, a favorable Tafel slope of 98 mV/dec, and the smallest semicircle diameter, indicating the lowest charge transfer resistance and enhanced electron transport efficiency. These findings confirm the synergistic enhancement realized through the hybridization of MOFs and g-C₃N₄, providing critical insights into the role of interfacial interactions in augmenting HER activity. The integration of theoretical and experimental methodologies in this research paves the way for the rational design of high-efficiency MOF-based photocatalysts, thereby advancing the development of green hydrogen technologies.

**Supplementary Information:**

The online version contains supplementary material available at 10.1038/s41598-025-20035-4.

## Introduction

The growing climate crisis, caused by greenhouse gas emissions and excessive fossil fuel consumption, is a significant threat to world sustainability. In response, the scientific world is increasingly focusing on renewable, low-carbon energy solutions, among which water splitting has gained recognition for its potential for generating clean hydrogen fuel utilizing abundant solar energy in an environmentally acceptable and cost-effective way. Hydrogen presents significant potential to enhance a clean, secure, and economically viable energy system. A primary advantage is its capacity to facilitate decarbonization by functioning as a clean and dependable method of energy storage and transportation^1–3^. This can diminish reliance on fossil fuels and bolster the resilience of energy systems against climate-induced disruptions. Following Environmental, Social, and Governance (ESG) principles, hydrogen is both ecologically sustainable and advantageous socially and economically. Moreover, it facilitates the attainment of multiple United Nations Sustainable Development Goals (SDGs), specifically SDG 7 (Affordable and Clean Energy), SDG 13 (Climate Action), and SDG 17 (Partnerships for the Goals)^4^. Hydrogen can be produced via several water-splitting mechanisms, including photocatalysis, electrolysis, photoelectrochemical (PEC) procedures, thermochemical processes, and biological methods^5–7^. All these methodologies substantially benefit from the presence of efficient catalysts, which lower activation energy barriers, boost selectivity, and accelerate the kinetics of hydrogen and oxygen evolution reaction^8,9^. Nonetheless, obstacles such as elevated costs, limited availability of active materials, inadequate long-term stability, and susceptibility to poisoning restrict the practical application of numerous conventional catalysts^10^.

Among the wide range of materials explored for the hydrogen evolution reaction (HER), (MOFs) and (g-C₃N₄) have emerged as promising photocatalytic and electrocatalytic candidates^11–13^. Zirconium-based metal-organic frameworks (MOFs), including UiO-66 and UiO-67, are esteemed for their remarkable stability and functionality^14,15^. Other notable types include MIL-series MOFs (e.g., MIL-53, MIL-101) characterized by substantial pore volumes, MOF-5 with a cubic zinc-based architecture, and HKUST-1 featuring copper paddlewheel units^16–18^. Moreover, MOF families such as IRMOFs and MOF-74 derivatives provide significant frameworks for investigating structure–property correlations and possess extensive applications in catalysis, gas storage, and drug delivery^19,20^. Precisely, UiO-66-NH₂ (U-N) is a zirconium-based (MOF) that possesses multiple characteristics suitable for hybrid catalytic systems^21–24^. It provides a substantial interior surface area (up to 1000 m²/g), facilitating homogeneous distribution of co-catalysts, conductive species, or metal nanoparticles. Its adjustable porosity, extensive surface area, and structural adaptability further augment its uses in catalysis, gas storage, and environmental cleanup. The amino-functionalized organic linkers in UiO-66-NH₂ facilitate post-synthetic alterations and enhance interactions with protons, guest molecules, and co-catalysts^25–27^. Additionally, the –NH₂ groups augment visible-light absorption and facilitate improved charge transport and interfacial interactions in composite systems. Furthermore, the Zr₆ clusters in UiO-66-NH₂ can coordinate with transition metals like Ni, Co, or Fe, facilitating the creation of metal-doped composites or single-atom catalysts with markedly enhanced catalytic activity^28^. However, MOFS’ performances and potential synergies vary significantly, and careful material selection and integration strategies are essential to optimize hydrogen generation^29–31^. When combined with conductive materials such as g-C₃N₄, graphene, or metal sulfides (e.g., MoS₂), UiO-66-NH₂ enhances charge separation and inhibits recombination under illumination, resulting in significant advancements in (HER) performance^32–34^. Moreover, Graphitic carbon nitride (g-C₃N₄), a metal-free polymeric semiconductor, has garnered interest due to its visible-light photoactivity, abundance in the Earth’s crust, and high nitrogen concentration. Nonetheless, the practical efficacy of pristine g-C₃N₄ is impeded by inadequate charge mobility and fast electron-hole recombination^35,36^. To address these limitations, researchers have created hybrid systems that integrate g-C₃N₄ with other materials to establish heterojunctions, facilitating enhanced charge separation and catalytic efficiency^37,38^.

The integration of UiO-66-NH₂ into heterostructures or hybrid composites leads to synergistic enhancements in performance. For example, in UiO-66-NH₂/g-C₃N₄ composites, the MOF serves as a porous matrix to anchor and stabilize the g-C₃N₄ network in both illuminated and non-illuminated environments, while also contributing to improved light harvesting and charge transfer^6,39,40^. Similarly, combinations with g-C₃N₄, Ni, Co, or Mo-based nanostructures can lower the overpotential, reduce the Tafel slope, and enhance hydrogen evolution kinetics^41–43^. Moreover, the incorporation of DFT calculations in recent research has proven invaluable for predicting band gaps, assessing charge distribution, and understanding reaction pathways at the molecular level^44,45^. Despite the considerable promise of (MOFs), particularly UiO-66-NH₂, in catalyzing the (HER), a comprehensive understanding of the mechanisms underlying their activity remains insufficient^46–48^. Although composites that incorporate co-catalysts like (g-C₃N₄) exhibit improved performance, it is often unclear whether this enhancement is attributable to the intrinsic properties of the MOF or synergistic interactions within the hybrid system^49,50^. This ambiguity highlights the necessity for thorough investigations that integrate experimental methodologies with first-principles computational techniques, such as DFT, to precisely evaluate band structure, charge transfer dynamics, and interfacial interactions. Such insights are crucial for the optimization of MOF-based materials for electrochemical and photo and electrocatalytic hydrogen production^51,52^.

This study presents a novel approach to the development of hydrogen evolution reaction (HER) catalysts by integrating thin-film morphology with precise composition tuning of UiO-66-NH₂ and g-C₃N₄ heterostructures, setting it apart from existing literature. Unlike many previous studies that focus solely on bulk materials or fixed compositions, our research emphasizes the optimization of weight ratios (60:40, 70:30, and 50:50) to achieve enhanced photocatalytic performance, demonstrating the impact of tailored compositions on electronic properties and catalytic activity. Furthermore, the incorporation of density functional theory (DFT) simulations alongside experimental methodologies provides a comprehensive framework for understanding the interfacial interactions and electronic structure, enabling a rational design strategy for high-efficiency MOF-based photocatalysts. This integrated approach not only elucidates the fundamental mechanisms underlying HER activity but also paves the way for future advancements in sustainable hydrogen production technologies. The paper adopts a multidisciplinary approach, combining synthetic strategies and experimental framework (Sect. 2), characterization results (Sect. 3), DFT analysis (Sect. 4), and results and discussion (Sect. 5). This integrated methodology is designed to elucidate the structure–property–performance relationships of the UiO-66-NH₂/g-C₃N₄ composites and to establish effective design strategies for developing high-performance, cost-effective catalysts for sustainable hydrogen production.

## Materials, synthetic strategies, and experimental framework

The following chemicals were used in this research without Further purification: 2-Aminoterephthalic acid (≥ 99%, Sigma-Aldrich), N,N-Dimethylformamide (DMF, ≥ 99.8%, for analysis EMSURE^®^ ACS, ISO, Reag. Ph Eur, Merck), Absolute Ethanol (≥ 99.9%, EMPLURA^®^, Merck), and Zirconium Tetrachloride (ZrCl₄, ≥ 99.5%, Loba Chemie, India) for the synthesis of UiO-66-NH₂. Melamine (2,4,6-Triamino-1,3,5-triazine, ≥ 99%, for synthesis, Merck) was used as the precursor for graphitic carbon nitride (g-C₃N₄). Other reagents included Acetone (≥ 99.5%, Merck), Methanol (≥ 99.8%, Merck), Glacial Acetic Acid (≥ 99.7%, Merck), Isopropanol (≥ 99.7%, Merck), and Potassium Hydroxide (KOH, ≥ 85%, Sigma-Aldrich). Sodium Sulfite (Na₂SO₃, ≥ 98%, Merck) was used as a hole scavenger during photoelectrochemical measurements. Substrates included microscopic glass slides and fluorine-doped tin oxide (FTO) glass.

**Fig. 1:**
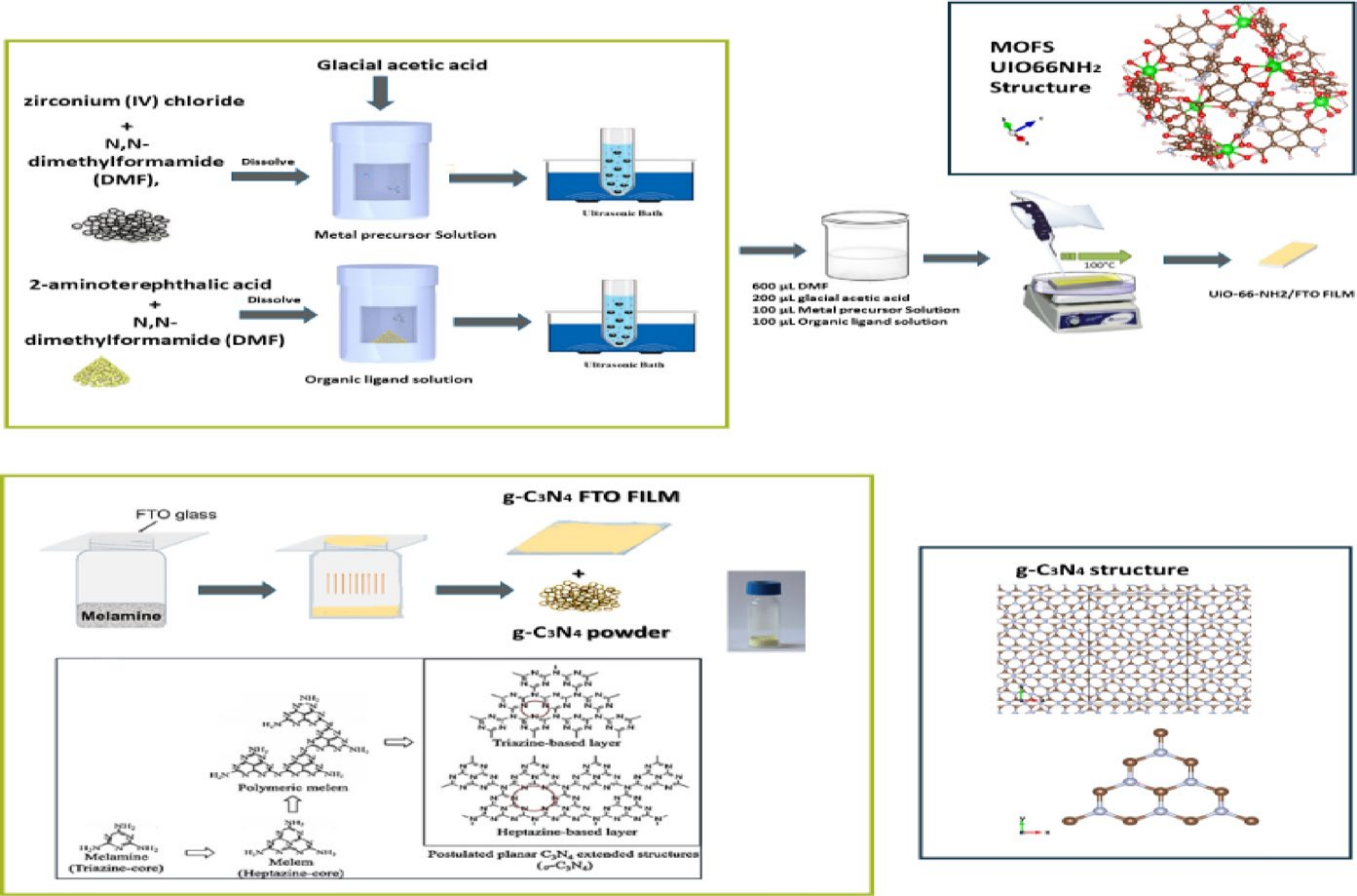
Synthesis of UiO-66-NH₂ films and g-C₃N₄ films/powder.

**Fig. 2:**
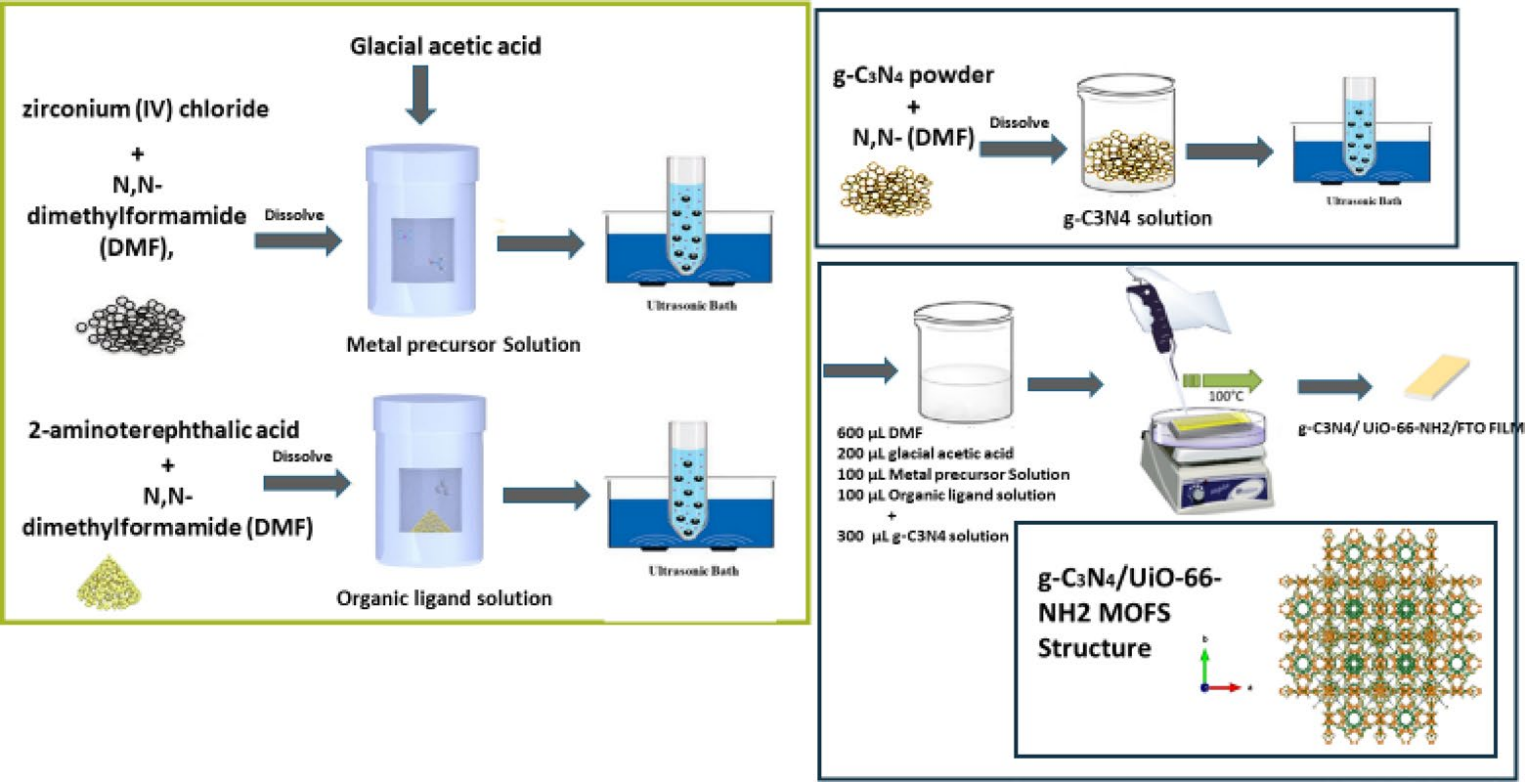
Preparation of g-C₃N₄)/UiO-66-NH₂ Films.

For thin film preparation, a modified sol-gel process was used to create thin films of the UiO-66-NH₂ on (FTO) surfaces. To facilitate the deposition process, clean glass microscope slides were placed within a Petri dish and heated to 100 °C on a hot plate, as shown in Fig. 1. Two precursor solutions were created in separate glass vials. To prepare the metal precursor solution, 175 mg of zirconium tetrachloride (ZrCl₄) was dissolved in 2.5 mL of N, N-dimethylformamide (DMF), then 0.5 mL of glacial acetic acid was added. To make the second solution with the organic Ligand, dissolve 182 mg of 2-aminoterephthalic acid in 2.5 mL of DMF and 0.15 mL of deionized water. To create the final casting solution, 600 µL of DMF, 200 µL of glacial acetic acid, and 100 µL of each metal ion and Ligand solution were mixed. The mixture was quickly mixed and put on a preheated substrate. The deposition was kept at 100 °C for 10 min beneath a glass cover to guarantee a uniform layer formation. To increase film thickness, many sequential castings were conducted before the previous layer was fully dried. This technique produced consistent thin yellow UiO-66-NH₂ films^53^. (g-C₃N₄) thin films were fabricated on FTO-coated glass slides. Before deposition, the FTO substrates were ultrasonically cleaned in acetone, ethanol, and deionized water for 15 min to ensure surface purity. The g-C₃N₄ films were synthesized using a thermal vapor condensation method as shown in Fig. 1. In this process, 7.0 g of melamine was placed inside a ceramic pot, and an FTO glass substrate was placed over the ceramic pot and covered with aluminum foil. The assembly was heated in a muffle Furnace at 520 °C for 4 h, using a temperature ramp rate of approximately 2 °C/min. After the reaction, the system was allowed to cool naturally to room temperature^54^. Synthesis of NH_2_-UiO-66 and g-C_3_N_4_/NH_2_-UiO-66 g-C_3_N_4_/NH_2_-UiO-66 was synthesized via different procedures reported in the past literature^41,43,50,55^. An altered sol–gel casting technique was utilized to synthesize g-C₃N₄/NH₂-UiO-66 composite thin films with different weight ratios on FTO and plain glass substrates. The composites were synthesized as deliberated in Fig. 2 in three distinct Weight ratios: 60 wt% NH₂-UiO-66/40 wt% g-C₃N₄, 70 wt% NH₂-UiO-66/30 wt% g-C₃N₄, and 50 wt% NH₂-UiO-66/50 wt% g-C₃N₄.

Before deposition, the glass substrates were meticulously cleaned and positioned on a hot plate set to 100 °C. The synthesis procedure commenced with the preparation of the various precursor solutions. To prepare the metal precursor, 175 mg of zirconium tetrachloride (ZrCl₄) was dissolved in 2.5 mL of N, N-dimethylformamide (DMF); thereafter adding 0.5 mL of glacial acetic acid was added. A Ligand solution was produced by dissolving 182 mg of 2-aminoterephthalic acid in 2.5 mL of DMF and 0.15 mL of deionised water. A g-C₃N₄ dispersion was made by ultrasonically dispersing 60 mg of g-C₃N₄ powder in 2.5 mL of DMF for 30 min to achieve homogeneity. To formulate the final casting solutions for each composition, the volumes of the g-C₃N₄ dispersion were modified to get the specified Weight percentages, while maintaining the other components at constant levels. Each mixture was swiftly agitated and promptly drop-cast onto the prepared substrate. The deposition technique was conducted at 100 °C for 10 min beneath a glass cover to guarantee uniform film formation. Numerous consecutive castings were executed while the preceding layer remained moist to increase the film thickness. This technique successfully produced consistent g-C₃N₄/NH₂-UiO-66 composite thin films with the desired weight ratios, rendering them appropriate for photocatalytic applications.

The synthesized materials were analyzed using a range of modern techniques to assess their structural, optical, morphological, and chemical properties. X-ray diffraction (XRD) patterns were obtained utilizing a Panalytical Empyrean 3 diffractometer (Malvern, Netherlands) to assess the crystallinity and phase purity of the materials. Optical absorption properties and band gap determinations were acquired by UV–Vis diffuse reflectance spectroscopy (DRS) utilizing a Cary 5000 UV–Vis–NIR spectrophotometer (Agilent Technologies, Malaysia). The surface morphology and internal microstructure were analyzed using field-emission scanning electron microscopy (FE-SEM GEMINI ULTRA 55) and transmission electron microscopy (JEOL JEM-2100), yielding comprehensive insights into particle dimensions, form, and composite structure. The surface elemental composition and chemical bonding states were examined via X-ray photoelectron spectroscopy (XPS) utilizing an AXIS Ultra system from Kratos Analytical Ltd.

## Photocatalytic and electrochemical performance assessments

Photocatalytic water splitting utilizes photons from solar irradiation to drive the hydrogen and oxygen evolution reactions, offering a sustainable, low-carbon pathway for hydrogen production. This light-driven process typically operates without the need for an externally applied electrical bias, relying instead on the semiconductor photocatalyst’s ability to absorb sunlight, generate charge carriers, and facilitate redox reactions at the surface. In contrast, electrochemical water splitting is entirely dependent on electrical energy supplied by an external source, such as a battery or power grid, and employs catalytic electrodes—commonly platinum or more abundant alternatives like nickel—to drive the reactions. To overcome kinetic Limitations and initiate the splitting process, a voltage exceeding the thermodynamic requirement of 1.23 V is needed, typically between 1.6 and 2.0 V due to overpotentials. While this method does not require light, coupling electrochemical systems with renewable energy sources like solar or wind power completes the green hydrogen production cycle, making both photocatalytic and electrochemical approaches integral to the advancement of clean energy technologies. Photoelectrochemical water splitting is a hybrid system that uses solar radiation in conjunction with a minimal external voltage to dissociate water molecules. It utilizes semiconductor photoelectrodes that absorb sunlight, producing photoexcited charge carriers (electrons and holes) that facilitate the reactions. In this research, photochemical and electrochemical mechanisms were tested.

Electrochemical measurements were conducted with a VersaSTAT4 Potentiostat/Galvanostat electrochemical workstation in a conventional three-electrode setup. A platinum wire functioned as the counter electrode (Cathode), a silver/silver chloride (Ag/AgCl, 3 M KCl) electrode served as the reference, and the working electrode (Anode/Photoanode) included FTO glass covered with the catalyst materials under testing. Before deposition, the FTO substrates were ultrasonically cleaned in acetone, ethanol, and deionized water for 15 min to ensure surface purity. The FTO substrates were linked to the workstation via insulated copper connectors to guarantee precise measurements. Three catalyst samples were evaluated^1^: (g-C₃N₄)^2^, the UiO-66-NH₂, and^3^composite materials consisting of UiO-66-NH₂ as the supporting substrate and (g-C₃N₄) as the dopant, made in three different weight ratios^1^: 0.6 UiO-66-NH₂/0.4 g-C₃N₄^2^, 0.7 UiO-66-NH₂/0.3 g-C₃N₄, and^3^ 0.5 UiO-66-NH₂/0.5 g-C₃N₄. These modifications seek to examine the impact of dopant concentration on the structural, optical, and catalytic characteristics of the hybrid materials. This configuration offered a regulated setting for assessing and contrasting the electrocatalytic efficacy of the synthesized materials concerning the hydrogen evolution process (HER). All electrochemical assessments were performed in a 1 M KOH aqueous electrolyte, produced by dissolving 5.8 g of potassium hydroxide (KOH) in 100 mL of deionized water and stirring for 15 min. Photocatalytic measurements were carried out in this study utilizing the same three-electrode cell design as the electrochemical testing, but with 0.5 M of Sodium Sulfite (Na_2_SO_3_) produced by dissolving 6.3 g of Sodium Sulfite (Na_2_SO_3_) in 100 mL of deionized water and stirring for 15 min as an electrolyte, with the addition of a solar simulator near the working electrode to simulate sunlight exposure and initiate the photocatalytic process.

To thoroughly assess the electrocatalytic performance of the synthesized materials for the HER, a range of electrochemical methods was utilized. The techniques employed comprised Linear Sweep Voltammetry (LSV), Tafel analysis, Cyclic Voltammetry (CV), and Electrochemical Impedance Spectroscopy (EIS). Polarization curves were obtained, and chronoamperometric (CA) testing was performed solely on the highest-performing sample to evaluate its long-term stability. All electrochemical potentials recorded in this work were originally measured relative to a silver/silver chloride (Ag/AgCl, 3 M KCl) reference electrode. To enable accurate comparison with standard literature values, these potentials were converted to the Reversible Hydrogen Electrode (RHE) scale by a two-step process. The measured potentials (E_meas) were referenced to the Standard Hydrogen Electrode (SHE) using the defined offset for the Ag/AgCl (3 M KCl) electrode, as per the following relation:

1 $$\:E\_SHE\:=\:E\_meas\:+\:0.210\:V$$

Following, the potentials were attuned to account for the pH of the electrolyte to obtain values relative to the RHE using the equation:

2 $$\:E\_RHE\:=\:E\_SHE\:+\:0.059\:\times\:\:pH$$

In 1 M KOH (pH 14), the entire conversion is simplified to:

3 $$\:E\_RHE\:=\:E\_meas\:+\:0.210\:+\:0.826\:=\:E\_meas\:+\:1.036\:V$$

This standard conversion ensures consistency in potential reporting in alkaline conditions and facilitates direct comparison with existing electrocatalytic data.

The overpotential (η) is a crucial parameter in the analysis of the hydrogen evolution process (HER), indicating the overpotential needed beyond thermodynamic equilibrium to facilitate the reaction at a specific current density. The calculation is performed using the formula:

4 $$\:\eta\:\:=\:E\_applied\:-\:E\_RHE$$

where E_applied is the experimentally measured potential and E_RHE is the reversible hydrogen electrode potential. Lower overpotentials correspond to better catalytic efficiency. Specifically, the potential value at a current density of −10 mA/cm² is considered, where the 0 V corresponds to the thermodynamic equilibrium potential:

5 $$\:\eta\:\:=\:0\:V\:at\:Potential\:(V\:vs.\:RHE)$$

The reaction kinetics of HER were further analyzed using Tafel plots, which relate the overpotential to the logarithm of the current density according to the Tafel equation:

6 $$\:\eta\:\:=\:b\:log\left(j\right)\:+\:a$$

where η is the overpotential, j is the current density, b is the Tafel slope, and a is the intercept. The Tafel slope provides important insights into the reaction mechanism and rate-determining step, with smaller slopes indicating faster kinetics and more favorable charge transfer characteristics.

## Characterization

**Fig. 3:**
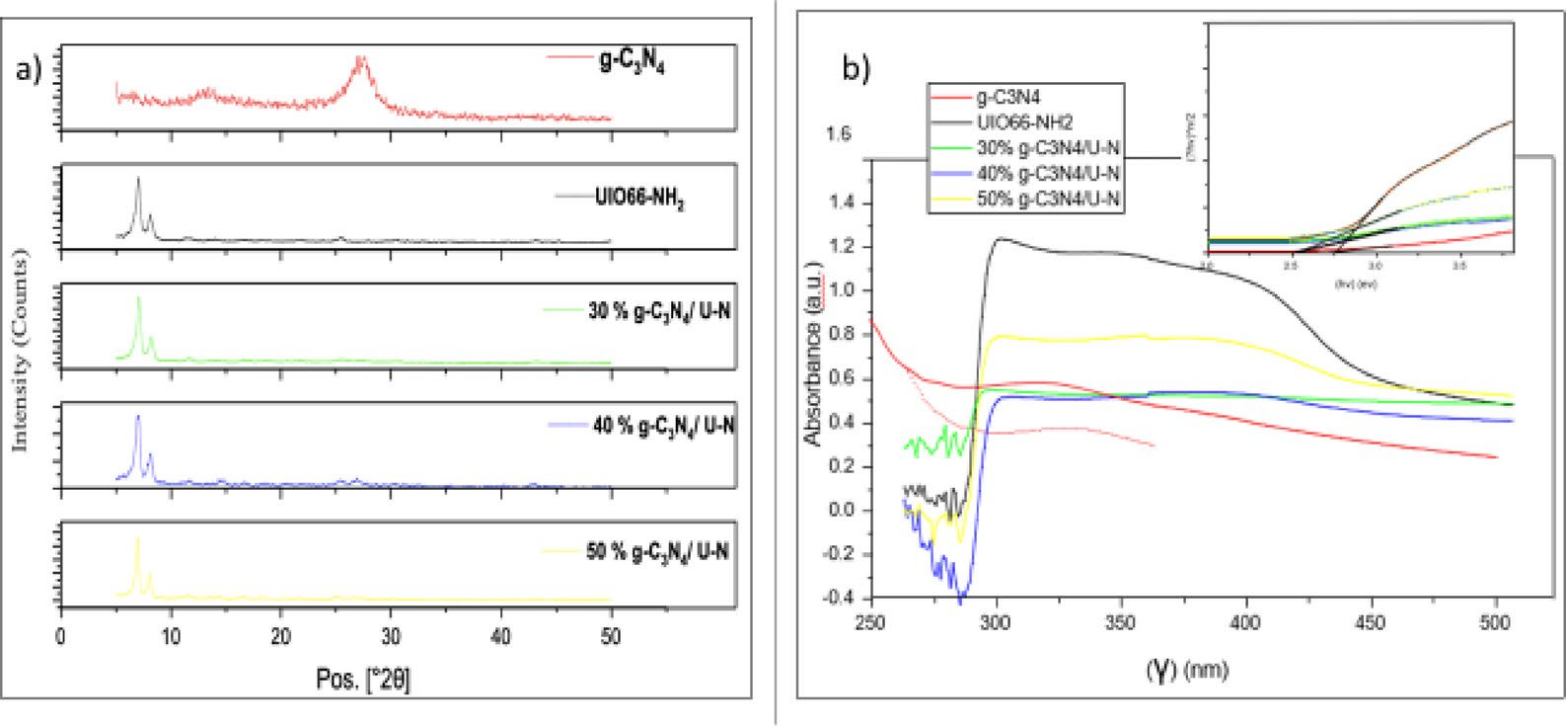
(a) X-ray diffraction (XRD) patterns of pure UiO-66-NH₂, g-C₃N₄, and a series of UiO-66-NH₂/g-C₃N₄ composites. (b) UV-Vis diffuse reflectance spectra and corresponding Tauc plots for the same materials. The band gap was determined by extrapolating the linear region of the (αhν)^(n/2)^ Vs hν plot to the x-axis, using *n* = 4 corresponding to an indirect allowed transition. Therefore, the XRD data support that the 30% g-C₃N₄ ratio offers the most favorable balance between crystallinity, dispersion, and interfacial interaction with UiO-66-NH₂, resulting in superior photo and electrocatalytic performance compared to the higher-loading composites. The progressive suppression of g-C₃N₄ peaks with higher loading indicates increased structural disorder and possible aggregation, which Likely hinders charge separation and reduces electron mobility. This aligns with the observed decline in photocatalytic performance at 40% and 50% g-C₃N₄ content, highlighting the importance of maintaining crystallinity and optimal dispersion for efficient hydrogen evolution.

The X-ray diffraction (XRD) patterns of pure UiO-66-NH₂, g-C₃N₄, and a series of UiO-66-NH₂/g-C₃N₄ composites are presented in Fig. 3-a. These patterns confirm the successful synthesis and structural integrity of the individual materials and their corresponding heterostructure, as they show that they are in good agreement with the simulated ones from single-crystal structure data. The XRD pattern of g-C₃N₄ aligns well with the conventional pattern catalogued under JCPDS No. 87-1526. It reveals two distinct diffraction peaks located at 13.43° and 27.19° (2θ), corresponding to the (100) and (002) planes of (g-C₃N₄), respectively. The peak at 13.43° with a d-spacing of 6.59 Å is attributed to the in-plane structural packing of tri-s-triazine (heptazine) units, indicating a well-ordered periodic arrangement within the layers. Meanwhile, the intense peak at 27.19°, exhibiting a d-spacing of 3.28 Å, is assigned to the interlayer stacking of the aromatic g-C₃N₄ sheets, reflecting strong π–π interactions between conjugated planes. These observations are in good agreement with literature values and confirm the successful formation of layered g-C₃N₄. The XRD pattern of the synthesized UiO-66-NH₂ displays characteristic peaks that confirm its successful crystallization. The most intense reflection is observed at 6.99° 2θ, corresponding to the (111) plane of the UiO-66 cubic framework, with a relative intensity of 100%, indicating a dominant crystallographic orientation. Other characteristic peaks appear at 8.15°, 11.60°, 13.88°, 16.79°, 18.40°, 21.66°, and 25.33° 2θ, which are assigned to the (002), (022), (113), (222), (024), (115), and (044) planes, respectively. These reflections align well with the standard pattern of UiO-66-type MOFs reported in the Literature, confirming the retention of the zirconium-based framework after amine Functionalization. Minor peaks at 27.80°, 29.61°, 30.43°, and 32.86° 2θ Likely correspond to higher-order reflections or framework vibrations. Also, the XRD pattern of the 30% g-C₃N₄/70% UiO-66-NH₂ composite demonstrates the coexistence of both crystalline phases, confirming successful hybrid material formation. A series of sharp diffraction peaks at 2θ values of 7.06°, 8.21°, 11.70°, and 16.52° is attributed to the (111), (002), (022), and (004) planes of the UiO-66-NH₂ framework, consistent with its well-known face-centered cubic topology. These reflections indicate that the integrity of the UiO-66-NH₂ crystalline structure is retained after composite formation. In addition, two characteristic peaks observed at 13.62° and 27.25° correspond to the (100) and (002) planes of (g-C₃N₄), associated with the in-plane packing of tri-s-triazine units and interlayer stacking of aromatic layers, respectively. The presence of these reflections confirms the successful incorporation of g-C₃N₄ into the UiO-66-NH₂ matrix and the formation of a hybrid material that maintains the structural features of both components. The XRD analysis supports the superior performance of the 30% g-C₃N₄/UiO-66-NH₂ composite, as evidenced by the well-resolved and intense diffraction peaks corresponding to both g-C₃N₄ and UiO-66-NH₂. Specifically, the 30% composite exhibits sharp peaks at 13.43° and 27.19° (2θ), which correspond to the (100) and (002) planes of g-C₃N₄, with relatively high relative intensities of 25.31% and 100%, respectively. These peaks reflect the preserved in-plane ordering and interlayer stacking of g-C₃N₄, indicating good crystallinity and minimal structural distortion. This well-ordered structure facilitates efficient π–π stacking interactions and enhances charge carrier mobility, which are critical for photo and electrocatalytic activity. In contrast, the 40% and 50% g-C₃N₄ composites show significantly weaker or broader g-C₃N₄ peaks. For instance, in the 40% sample, the (002) peak at 27° becomes much less intense (only 2.08% relative intensity), and the (100) peak is barely visible. In the 50% composite, although additional peaks appear due to higher g-C₃N₄ content, they are broader and less defined, suggesting increased disorder and possible agglomeration. This structural disruption likely hampers charge transport and reduces active surface area, leading to diminished performance.

The UV-Vis diffuse reflectance spectra provide insight into the optical absorption properties of pure g-C₃N₄, UIO-66-NH₂, and their composites. In Fig. 3-b, pure g-C₃N₄ shows strong absorption in the UV and visible regions, with a sharp onset of absorption at around 450 nm, indicating its efficacy in harvesting visible light. UIO-66-NH₂, on the other hand, exhibits two distinct absorption features: a sharp peak around 250 nm, attributed to O→Zr charge transfer transitions within the Zr₆-oxo cluster, and a broader peak between 320 and 380 nm, corresponding to ligand-to-metal charge transfer (LMCT) involving the amino-functionalized organic linker. As g-C₃N₄ is introduced into the UIO-66-NH₂ framework (composite samples with 30%, 40%, and 50% g-C₃N₄ content), the resulting spectra show broader absorption profiles extending into the visible region (up to 450 nm), consistent with the light-harvesting characteristics of g-C₃N₄. With increasing g-C₃N₄ content, the absorption edge shifts slightly toward longer wavelengths (redshift), implying enhanced visible-light utilization in the composites. The optical band gaps of pure g-C₃N₄, UIO-66-NH₂, and their hybrid composites were determined using Tauc plot analysis, assuming indirect electronic transitions. The band gap energy was calculated using Tauc plot analysis based on the following equation:

8 $$\:{\left(\alpha\:h\nu\:\right)}^{\frac{n}{2}}{=A(h\nu\:-Eg)}^{\frac{n}{2}}$$

where α is the absorption coefficient, h is Planck’s constant, ν is the photon frequency, A is a proportionality constant, and n is a parameter determined by the optical transition type of the semiconductor. For direct band gap transitions, *n* = 1, whereas for indirect transitions, *n* = 4. The band gap was obtained by extrapolating the linear region of the $\:{\left(\alpha\:h\nu\:\right)}^{\frac{n}{2}}$ Vs hν plot to the x-axis. In this study, a value of *n* = 4 was used, indicating the assumption of an indirect band gap transition.

In Fig. 3-b, pure g-C₃N₄ exhibited a band gap of approximately 2.75 eV, while UIO-66-NH₂ showed a slightly larger band gap of 2.83 eV, consistent with its wide-gap semiconductor nature and limited visible light absorption. Interestingly, upon forming g-C₃N₄/UiO-66-NH₂ composites, a notable reduction in band gap energy was observed, with the 30% g-C₃N₄ composite showing the lowest band gap (2.68 eV). The 40% and 50% composites exhibited slightly higher values of 2.72 eV and 2.70 eV, respectively. This decrease in band gap upon composite formation is not simply a weighted average of the two materials but rather reflects synergistic electronic interactions at the g-C₃N₄/UiO-66-NH₂ interface. The coupling of these two materials leads to a modification in the electronic band structure, attributed to the formation of heterojunctions and possible band bending at the interface. Such changes enhance charge transfer across the junction and lower the energy required for electronic excitation. Firstly, interfacial electronic interactions between g-C₃N₄ and UiO66-NH₂ induce band bending and favorable band alignment, facilitating efficient charge separation at the junction. The conduction band minimum (CBM) of g-C₃N₄ is typically around –0.3 V vs. NHE, while the valence band maximum (VBM) is near + 1.4 V vs. NHE. In comparison, UiO-66-NH₂ generally exhibits a higher-lying VBM and a slightly less negative CBM.

This band alignment creates a staggered (Type-II) heterojunction, where electrons can migrate from g-C₃N₄ to UiO-66-NH₂ and holes move in the opposite direction, thus reducing recombination rates. The conduction band minimum (CBM) of g-C₃N₄ is typically around –0.3 V vs. NHE, while the valence band maximum (VBM) is near + 1.4 V vs. NHE. In comparison, UiO-66-NH₂ generally exhibits a higher-lying VBM and a slightly less negative CBM. The flat-band potentials were − 1.00 V for g-C₃N₄, − 0.65 V for UiO-66-NH₂, and − 0.80 V for the 30% g-C₃N₄/UiO-66-NH₂ composite (vs. Ag/AgCl), shown in the Supplementary Materials, Figure S1. Converting to the NHE scale and combining with the respective band gaps (g-C₃N₄ ≈ 2.75 eV, UiO-66-NH₂ ≈ 2.83 eV, and the composite ≈ 2.63 eV), the conduction and valence band positions confirm a staggered arrangement, where the CB of g-C₃N₄ is more negative and the VB of UiO-66-NH₂ is more positive. This staggered alignment enables spatial separation of electrons and holes, which is characteristic of a Type-II heterojunction. Secondly, orbital hybridization between the π-conjugated structure of g-C₃N₄ and the amino-functionalized linkers in UiO-66-NH₂ may result in mid-gap states or band tailing, lowering the photon energy required for excitation. Secondly, orbital overlap between the conjugated structure of g-C₃N₄ and the amino-functionalized organic linkers in UiO-66-NH₂ may create mid-gap states or extend the tail of the conduction/valence bands, effectively reducing the optical band gap. Thirdly, this structure ensures that the band edge positions remain suitable for redox reactions, with the CBM still negative enough for proton reduction and the VBM positive enough for oxidation reactions, such as water oxidation or organic pollutant degradation. The composite structure promotes efficient charge separation by enabling directional migration of electrons and holes across the heterojunction, thereby suppressing charge recombination, a major limiting factor in photocatalysis. Lastly, the structural integration enhances visible-light harvesting and promotes electron–hole pair dissociation, all of which contribute to the superior photocatalytic performance of the composite materials compared to their pristine counterparts.

**Fig. 4:**
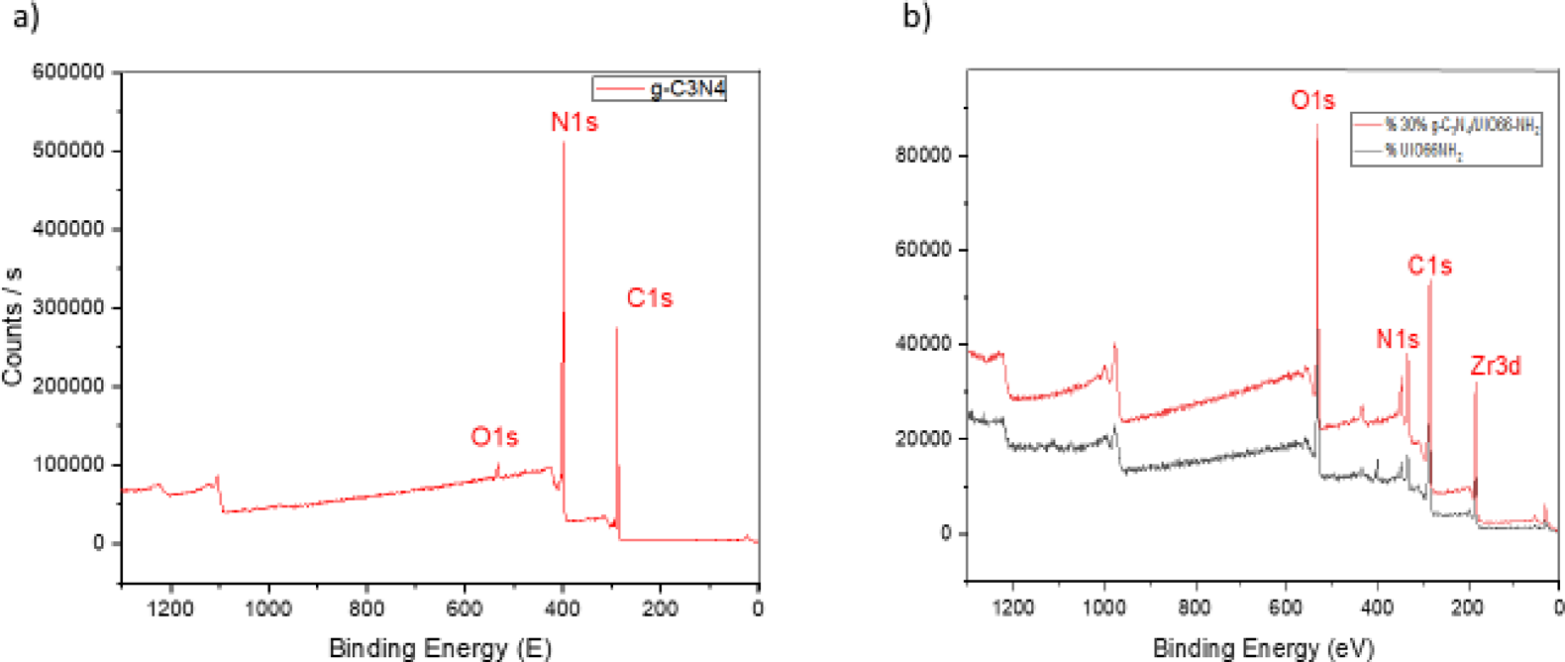
X-ray Photoelectron Spectroscopy (XPS) survey spectra showing the energy states of the main elements in (a) pure UiO-66-NH₂, and (b) pure g-C₃N₄ and 30 wt% g-C₃N₄/UiO-66-NH₂ composite. The patterns identify the binding energies associated with key elements, providing insight into their chemical states and surface composition.

**Fig. 5:**
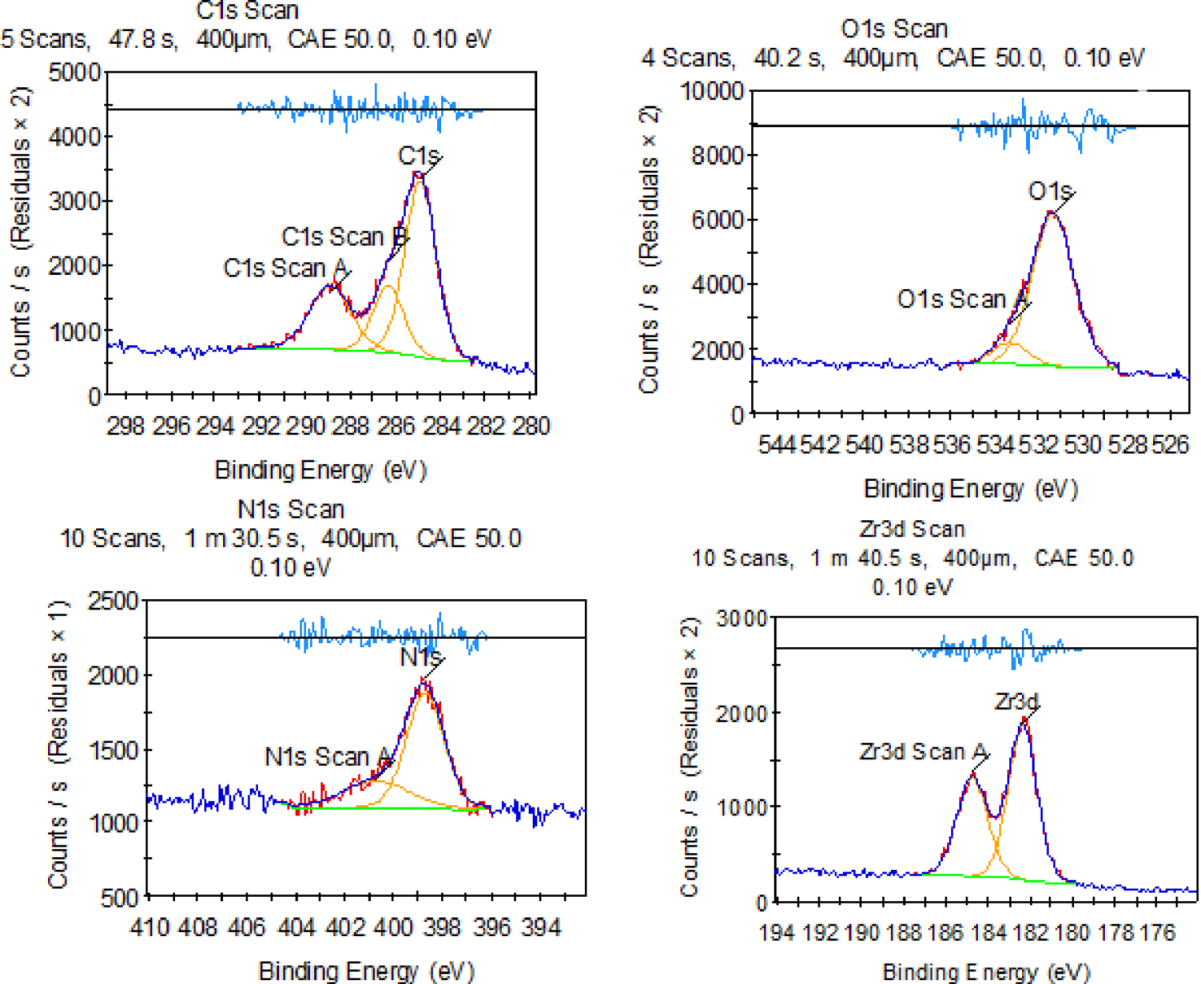
Regional XPS spectra of C 1 s, N 1 s, O 1 s, and Zr 3 d of 30 wt% g-C₃N₄/UiO-66-NH₂ composite.

X-ray photoelectron spectroscopy (XPS), as shown in Fig. 4, was employed to provide information on the chemical Environment and to examine the energy states of the pure substances: (g-C₃N₄) and UIO-66-NH₂. The XPS spectrum of pure g-C₃N₄ reveals significant peaks at C 1 s (285 eV), N 1 s (400 eV), and a notable O 1 s peak (531 eV), presumably attributed to adsorbed surface oxygen or hydroxyl molecules. The detection of Zr 3 d peaks is unexpected in g-C₃N₄, suggesting possible minor contamination or substrate effects. The prominent N 1 s and C 1 s peaks validate the nitrogen-enriched heptazine-derived structure of g-C₃N₄, which is crucial to its photocatalytic properties. The XPS spectrum of UIO-66-NH₂ has pronounced peaks for N 1 s, C 1 s, and O 1 s, indicative of the amino-functionalized organic linker (2-aminoterephthalic acid) and the zirconium-based metal cluster. The prominent N 1 s peak (400 eV) signifies the existence of amino groups, whereas the O 1 s peak (531 eV) arises from both carboxyl groups and Zr–O bonds in the metal-organic framework. The lack of Zr 3 d peaks in this broad scan may result from instrumental sensitivity or surface termination effects; however, the characteristic peaks validate the anticipated elemental composition of UIO-66-NH₂0.30% g-C₃N₄/UiO-66-NH₂ composite confirms the successful incorporation of all expected elements within the heterostructure. Distinct peaks corresponding to O 1 s (~ 530 eV), C 1 s (~ 285 eV), N 1 s (~ 400 eV), and Zr 3 d (~ 182–185 eV) are observed. The presence of Zr 3 d confirms the retention of the zirconium-based metal clusters from the UiO-66-NH₂ framework. The O 1 s and C 1 s peaks are attributed to the organic linkers in the MOF, surface-adsorbed species (e.g., –OH, H₂O), and g-C₃N₄. The prominent N 1 s signal is particularly indicative of the high nitrogen content introduced by both the amine-functionalized UiO-66 and the g-C₃N₄ matrix, validating the successful surface integration of the nitrogen-rich graphitic carbon nitride. Collectively, the elemental composition revealed by XPS supports the formation of a well-defined heterojunction between UiO-66-NH₂ and g-C₃N₄, which is essential for enhancing the photo- and electrocatalytic performance of the composite. In addition to the regional XPS spectra of C 1 s, N 1 s, O 1 s, and Zr 3 d of the 30 wt% g-C₃N₄/UiO-66-NH₂ composite, are shown in Fig. 5.

**Fig. 6:**
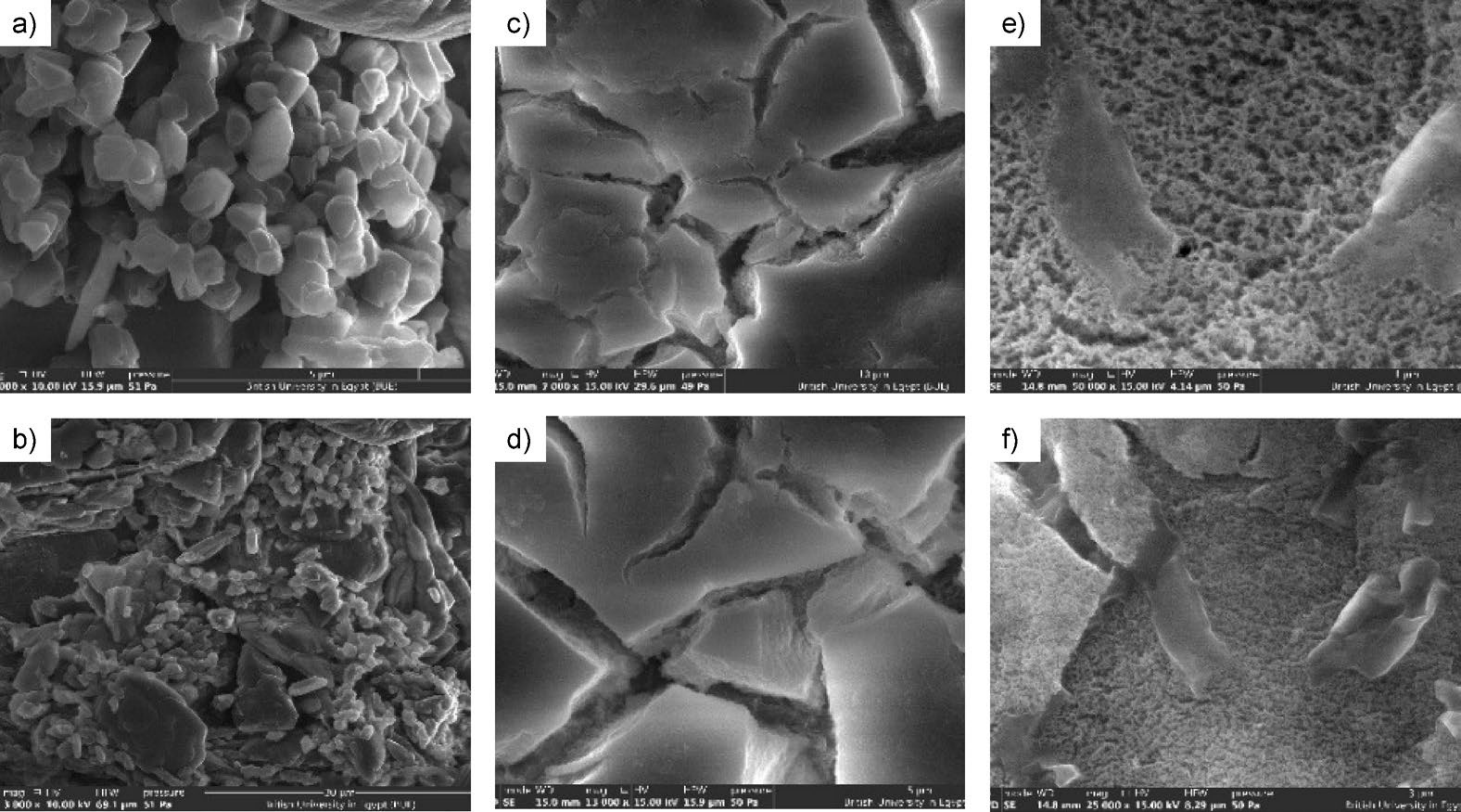
Scanning Electron Microscopy (SEM) micrographs of (a), (b) pure g-C₃N₄, (c), (d) pure UiO-66-NH₂, and (e), (f) the 30 wt% g-C₃N₄/UiO-66-NH₂ composite.

The SEM micrographs provide comprehensive insight into the morphological characteristics of g-C₃N₄, UiO-66-NH₂, and their 30% g-C₃N₄/UiO-66-NH₂ composite, shown in Fig. 6. The g-C₃N₄ sample, depicted in (a, b), exhibits a layered and agglomerated morphology, consisting of loosely packed sheets and irregularly shaped particles. This structure is characteristic of graphitic carbon nitride and signifies a high surface area, advantageous for catalytic applications because of enhanced exposure of active sites. These sheets demonstrate an extensive π-conjugated system that enhances the semiconducting characteristics of g-C₃N₄, rendering it appropriate for photocatalysis. Conversely, the UiO-66-NH₂ material, depicted in (c, d), has a distinct octahedral or pseudo-cubic crystalline shape, marked by uniform particle distribution, smooth surfaces, and sharp edges. These characteristics validate the elevated crystallinity and resilient structural framework of the Zr-MOF. The occurrence of fissures or cavities among certain crystals may result from solvent evaporation or structural contraction during sample preparation, a prevalent feature in MOF materials. The composite sample, comprising 30% g-C₃N₄ with UiO-66-NH₂ (e, f), demonstrates a distinct incorporation of g-C₃N₄ nanosheets within and atop the MOF crystals. The course, a more heterogeneous structure of the composite, indicates that g-C₃N₄ is adequately disseminated and securely affixed to the UiO-66-NH₂ surface. This hybrid morphology signifies effective interfacial contact, perhaps via hydrogen bonding or π–π stacking between the nitrogen-rich planes of g-C₃N₄ and the amino functional groups of the MOF. The resultant structure integrates the elevated porosity and chemical stability of UiO-66-NH₂ with the photocatalytic efficacy and electronic conductivity of g-C₃N4.

**Fig. 7:**
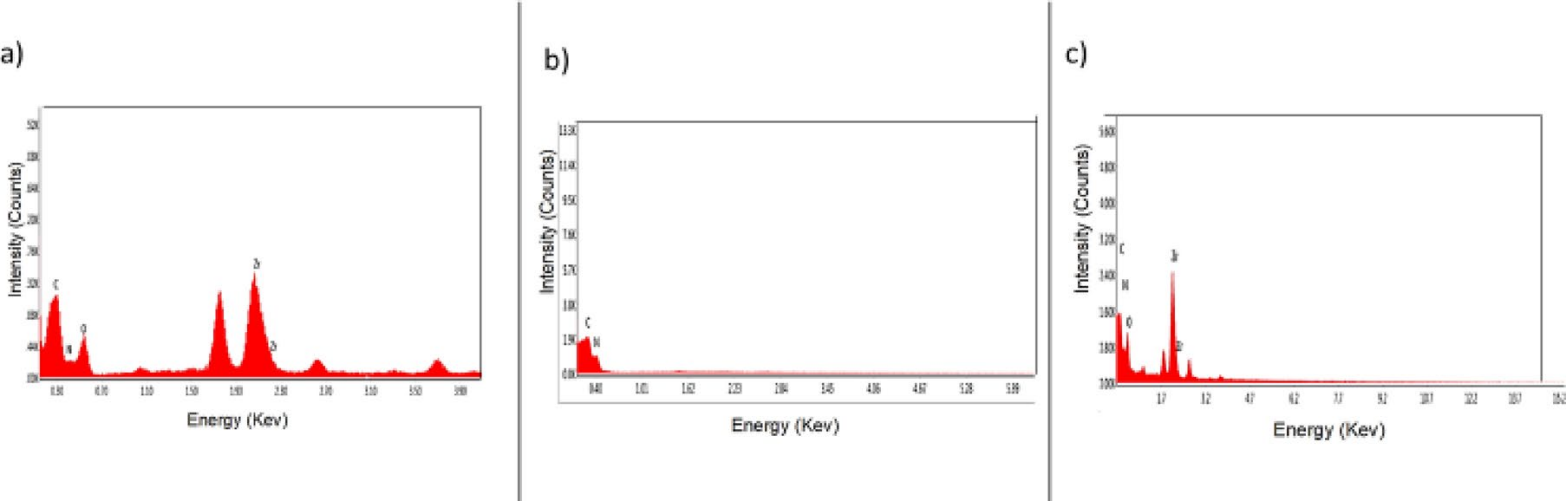
Energy-dispersive X-ray (EDX) analysis of the proposed materials: (a) pure g-C₃N₄, (b) pure UiO-66-NH₂, and (c) the 30 wt% g-C₃N₄/UiO-66-NH₂ composite.

The energy-dispersive X-ray spectroscopy (EDX) analysis was conducted to investigate the elemental composition of UiO-66-NH₂, g-C₃N₄, and their corresponding composite (30% g-C₃N₄/UiO-66-NH₂), shown in Fig. 7. The EDX spectrum of UiO-66-NH₂ (a) revealed prominent peaks corresponding to carbon (C), nitrogen (N), oxygen (O), and zirconium (Zr), which confirms the successful synthesis of the metal-organic framework, with Zr representing the metal node and C, N, and O originating from the amino-terephthalic acid linker and coordinated oxygen species. In contrast, the g-C₃N₄ spectrum (b) displayed only C and N peaks, consistent with its polymeric structure based on tri-s-triazine units, and the absence of oxygen or metal peaks Further verified its purity. The EDX profile of the 30% g-C₃N₄/UiO-66-NH₂ composite (c) showed the presence of all characteristic elements (C, N, O, and Zr), indicating successful integration of g-C₃N₄ into the UiO-66-NH₂ framework. Notably, the increased intensity of C and N peaks in the composite spectrum relative to the pristine UiO-66-NH₂, along with a moderate reduction in Zr signal, supports the effective incorporation of g-C₃N₄ and confirms the formation of the hybrid material.

**Fig. 8:**
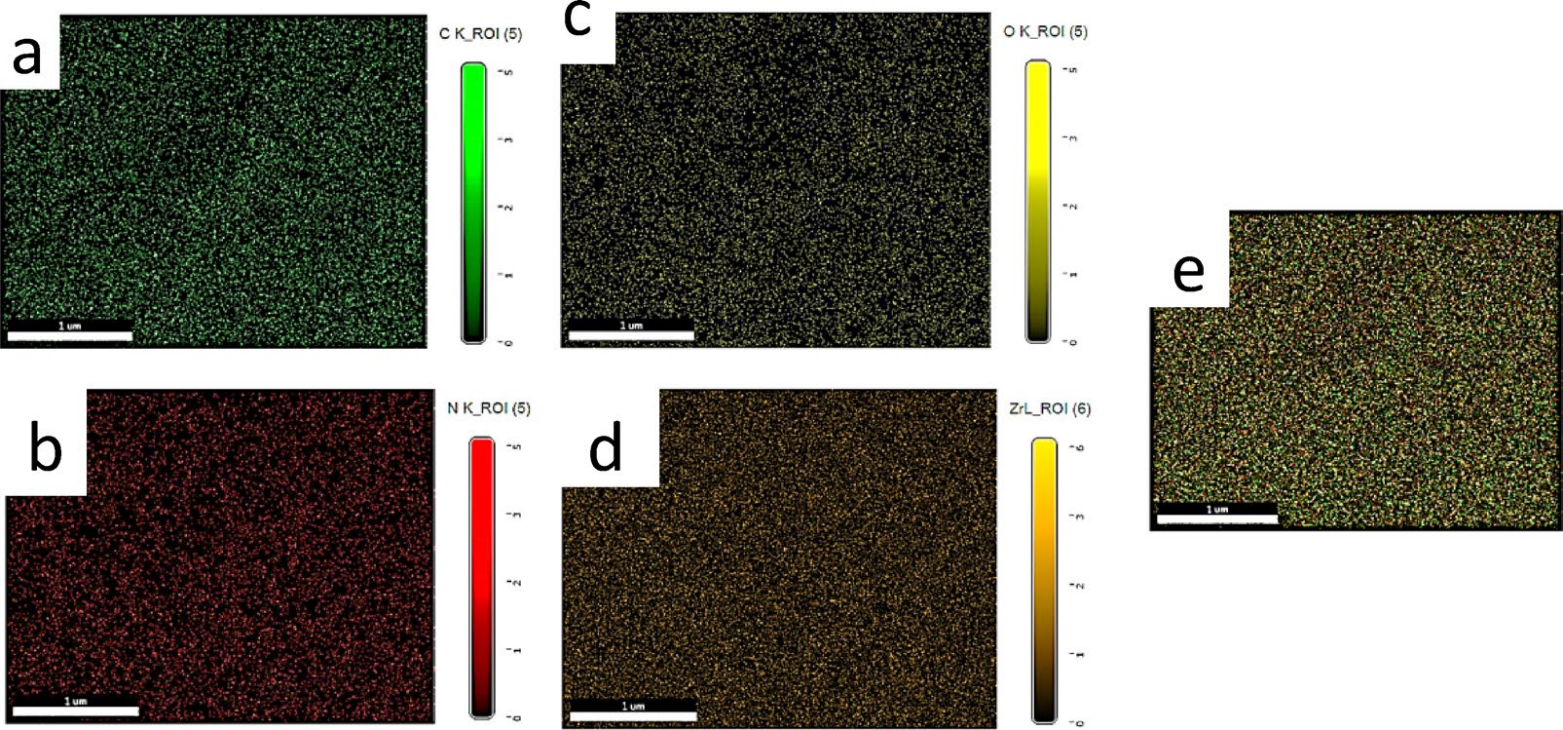
The elemental mapping of the 30 wt% g-C₃N₄/UiO-66-NH₂ composite obtained by energy-dispersive X-ray spectroscopy (EDS). (a) C Carbon, (b) N Nitrogen, (c) O Oxygen, (d) Zr Zirconium, (e) 30 wt% g-C₃N₄/UiO-66-NH₂.

The elemental mapping of the 30% g-C₃N₄/UiO-66-NH₂ composite, obtained through EDS, shown in Fig. 8, indicates a uniform and well-dispersed distribution of the key constituent elements: carbon (C), nitrogen (N), oxygen (O), and zirconium (Zr). Carbon, depicted in green, is uniformly distributed across the surface, validating the consistent integration of both the g-C₃N₄ nanosheets and the organic linkers within the UiO-66-NH₂ framework. Nitrogen, seen in red, is consistently distributed, though at a marginally reduced intensity, indicating the presence of nitrogen-rich g-C₃N₄ and amino functional groups inside the MOF. The yellow-hued oxygen mapping demonstrates uniform dispersion, ascribed to the carboxylate groups in UiO-66-NH₂ and potential surface oxygen functions. Zirconium, represented in orange, displays a robust and consistent signal throughout the sample region, confirming the structural integrity of the Zr-based UiO-66-NH₂. The elemental mapping verifies the successful creation of a uniformly integrated composite, with evenly dispersed g-C₃N₄ within the UiO-66-NH₂ matrix, which is crucial for improving the material’s photocatalytic performance, charge transfer, and structural stability.

## DFT analysis

Quantum ESPRESSO was employed to investigate the electronic structure of g-C₃N₄ monolayer, pristine UiO-66, amino-functionalized UiO-66-NH₂, and the composite UiO-66-NH₂/g-C₃N₄. Besides complementing the theoretical and computational setup, a full density function theory (DFT) input files were constructed for predicting key properties like band structure, density of states (DOS), and charge distribution, critical for evaluating photocatalytic performance. The crystallographic models of UiO-66-NH₂, g-C₃N₄, and their hybrid were constructed and validated for periodicity and symmetry using VESTA software, with atomic coordinates exported to Quantum ESPRESSO.

(DFT) is based on the theorems of Hohenberg and Kohn, which assert that the energy of a chemical system is directly determined by the ground-state electron density. The Kohn − Sham approach is employed to derive the ground-state electron density of interacting electrons inside a static external potential, utilizing density functions that represent individual, noninteracting electrons. The principal limitation of DFT is the absence of a known functional that complies with the Hohenberg-Kohn theorem, hence precluding the computation of the exact energy or ground-state density of a system. A broad range of functionals is available, differing in their approximate treatment of the exchange-correlation (XC) term of the Hamiltonian, where exchange and correlation represent purely quantum mechanical effects, conceptualized as repulsive and attractive terms, respectively. The challenge is to determine which functional yields the most accurate electron density or energy for a specific chemical system, based on the parameters utilized in functional construction and the prevailing physics in the MOF. Given the absence of an exact XC functional, approximations were necessary, categorized hierarchically (“Jacob’s Ladder”), the Local Density Approximation (LDA) for uniform densities (e.g., metals), the Generalized Gradient Approximation (GGA) incorporating density gradients (e.g., PBE functional), and costlier hybrid functionals (e.g., HSE06) for improved band gaps.

In this study, the PBE functional was selected to balance computational tractability and reliability for the MOF-semiconductor heterostructure, though its tendency to underestimate band gaps was accounted for in interpreting results. All systems were analyzed using the spin-polarized self-consistent field (SCF) approximation, wherein the Kohn–Sham equations are solved iteratively until convergence of the total energy is achieved. Pseudopotentials and plane-wave basis sets were employed to describe electron-ion interactions, ensuring an efficient yet accurate representation of the electronic structure.

**Fig. 9:**
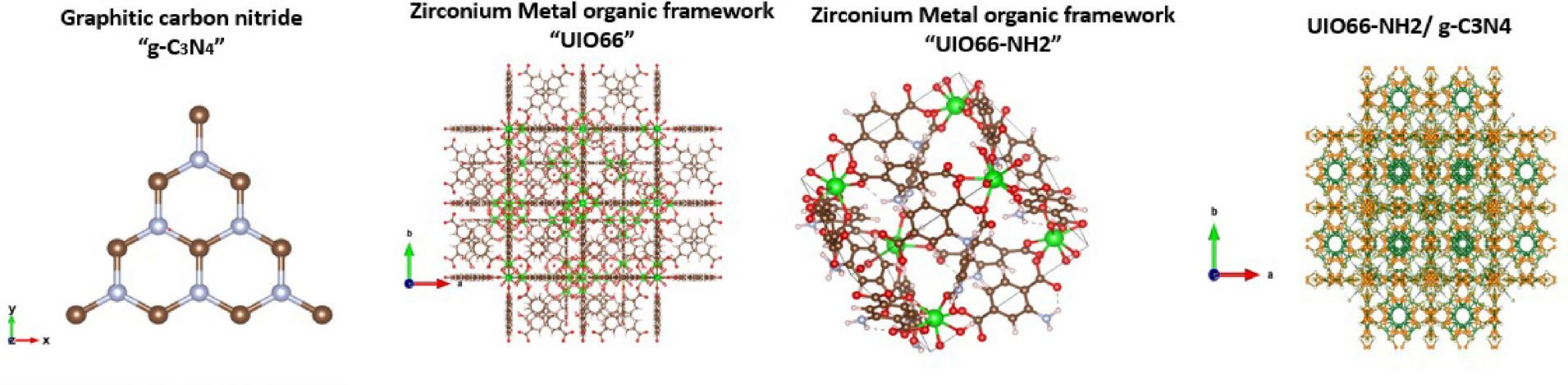
Visual representation of the crystal structures generated using VESTA software: pure UiO-66, pure UiO-66-NH₂, pure g-C₃N₄, and the proposed UiO-66-NH₂/g-C₃N₄ composite.

As visualized in Fig. 9, the UiO-66 and UiO-66-NH₂ (MOFs) both crystallize in cubic symmetry (space group *Fm-3 m*), with Zr₆O₄(OH)₄ clusters as 12-connected inorganic nodes bridged respectively by terephthalic acid (BDC) and 2-aminoterephthalic acid (BDC-NH₂) linkers. UiO-66 provides high porosity and structural stability but lacks functional groups on the benzene ring, limiting its chemical tunability, whereas UiO-66-NH₂ introduces –NH₂ groups, enhancing reactivity and molecular interactions. Atomically, Zr (green), O (red), C (brown), and H (Light Pink) define the backbone of both frameworks, with N (purple) present only in the amino-functionalized variant. In contrast, (g-C₃N₄) features a 2D layered structure composed of tri-s-triazine (heptazine) units interconnected by nitrogen bridges (C: *N* ≈ 3:4), where C (brown) and N (Purple) occupy the ring and bridging positions, respectively. The layers stack via van der Waals forces, resembling graphite, and provide visible-light photocatalytic activity due to a moderate band gap and efficient charge mobility.

The UiO-66-NH₂/g-C₃N₄ heterostructure integrates both materials to create a hybrid interface, utilizing the porosity and active sites of the MOF in conjunction with the photo-responsiveness of g-C₃N₄. The contact probably entails hydrogen bonding (between –NH₂ and g-C₃N₄’s nitrogen sites) or van der Waals forces, enabled by a supercell design to alleviate lattice mismatch. This design seeks to improve charge separation and photocatalytic efficiency, rendering it advantageous for energy conversion and environmental remediation applications. The g-C₃N₄ system, represented as a layered two-dimensional material, was characterized using a hexagonal lattice and simulated with a 6 × 6 × 1 Monkhorst-Pack k-point grid with a kinetic energy cutoff of 30 Ry. For the extensive MOF systems, such as UiO-66 and UiO-66-NH₂, spin-polarized computations utilized elevated cutoffs (60 Ry for wavefunctions and 480 Ry for charge density), cold smearing, and meticulously designated initial magnetic moments to accurately capture nuanced electronic and magnetic characteristics. The composite UiO-66-NH₂/g-C₃N₄ system, comprising more than 800 atoms, was analyzed utilizing a completely relaxed triclinic cell with a 2 × 2 × 2 k-point grid and convergence thresholds refined for precision in extensive systems. The input configurations were rigorously validated to guarantee structural integrity, numerical stability, and significant electronic outputs, establishing a solid basis for analyzing charge redistribution, interfacial electronic alignment, and photocatalytic potential at the MOF/2D material interface.

For the k-point sampling, a 6 × 6 × 1 Monkhorst-Pack grid was employed for the g-C₃N₄ system, which, being a two-dimensional layered material, required a denser grid along the z-direction to adequately capture the electronic behavior within its layered architecture while minimizing computational resource demands. This grid resolution ensures a reliable integration of the Brillouin zone, accommodating the periodicity of the system and thereby providing a more accurate representation of the band structure and density of states. For the larger MOF systems, including UiO-66 and UiO-66-NH₂, higher energy cutoffs were chosen (60 Ry for wavefunctions and 480 Ry for charge density) to enhance the precision of the calculated electronic properties. These cutoffs are essential for ensuring that the plane-wave basis sets adequately describe the fast-evolving electronic states, especially in systems with heavy transitional metal centers like zirconium.

## Results and discussions

In this section, all the reported results, including theoretical DFT investigations, photochemical analysis, and electrochemical reactions, are illustrated.

### DFT results

**Fig. 10:**
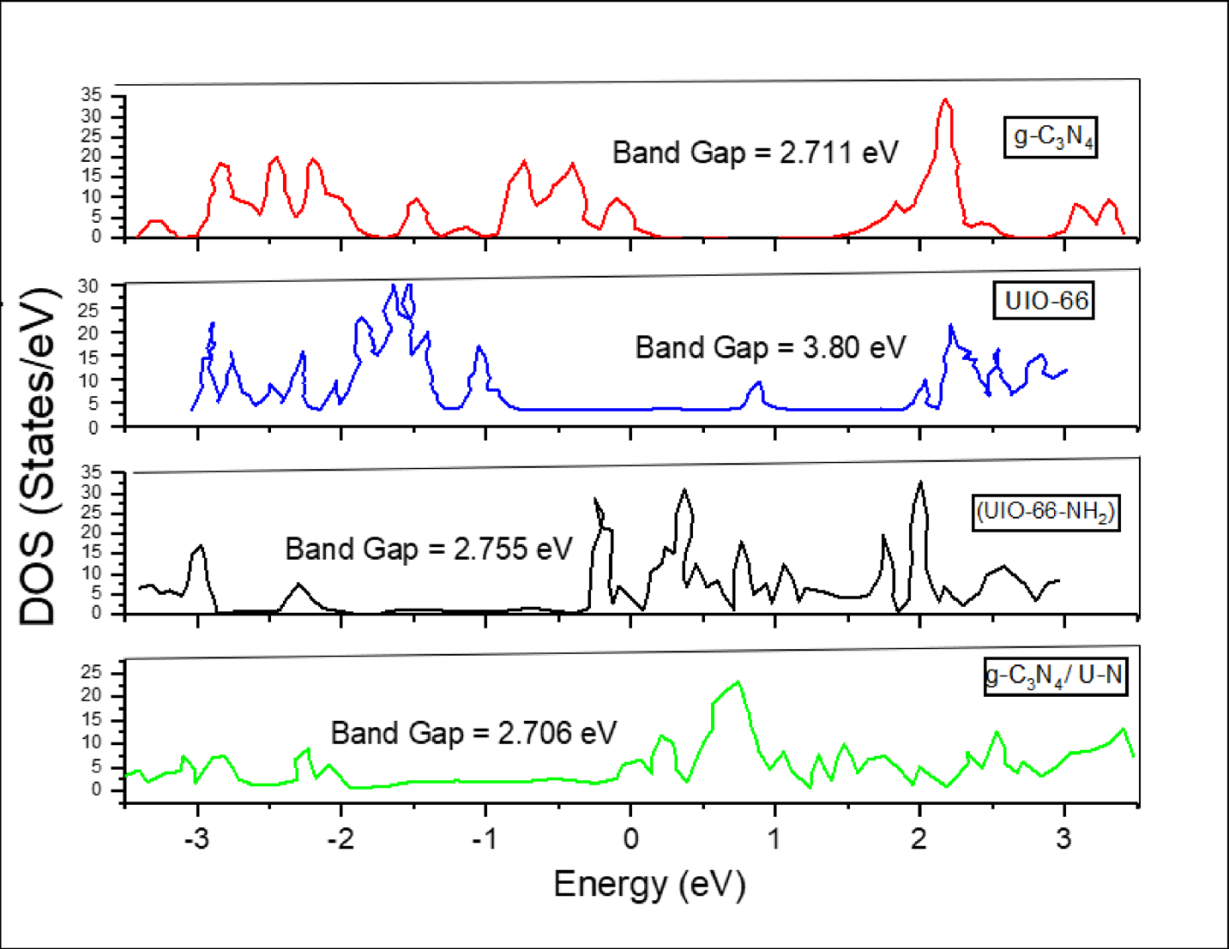
(DOS) and band gap of the proposed materials—pure UiO-66-NH₂, pure g-C₃N₄, and the UiO-66-NH₂/g-C₃N₄ composite—obtained from (DFT) calculations using Quantum ESPRESSO.

The electronic band structures and (DOS) were analyzed for four composites to evaluate their suitability for photocatalytic hydrogen production: pristine g-C₃N₄, and UiO-66 as bare structures, in addition to amino-functionalized UiO-66-NH₂, and the g-C₃N₄/UiO-66-NH₂ composites, see illustrated in Fig. 10. Pristine UiO-66 exhibits a wide bandgap (3.8 eV), limiting visible-light absorption, and Pristine g-C₃N₄ (2.711 eV). While the incorporation of the electron-donating amino group in UiO-66-NH₂ markedly decreases the bandgap, UiO-66-NH₂ shows a reduced bandgap (2.755 eV) due to nitrogen-induced mid-gap states near the valence band maximum (VBM), enhancing charge mobility and light absorption.

The composite shows a reduced band gap of around 2.706 eV, when these two materials are combined, their complementary band edge positions facilitate efficient charge separation: photoexcited electrons can transfer from the conduction band of g-C₃N₄ to that of UiO-66-NH₂, while holes migrate in the opposite direction. This heterojunction structure effectively suppresses electron-hole recombination and broadens the light absorption range. Therefore, the UiO-66-NH₂/g-C₃N₄ composite exhibits great promise for enhanced photocatalytic hydrogen production under visible light. To minimize experimental duration and expenses, we began with the DFT simulations to evaluate the electrical characteristics of virgin UiO-66 and amine-functionalized UiO-66-NH₂. The findings indicated that amine functionalization significantly lowered the bandgap and enhanced band alignment for water splitting. Consequent on these findings, we identified UiO-66-NH₂ as the more appropriate candidate for experimental fabrication for HER. Computational findings identified g-C₃N₄/UiO-66-NH₂ heterojunction as the most promising system for enhanced photocatalytic hydrogen evolution.

To ensure the variability of the DFT model, we accredited the exported electrochemical, charge transfer mechanism, and optical properties for both bare and composite structures against similar experimental investigations in the literature, see citation list in Table 1. The theoretical results showed very good agreement with the experimental data in terms of bandgap calculations as well as absorption spectra. This can validate our DFT model capabilities to describe the bare structure as well as the MOFs campsites. The charge transfer analysis (e.g., Bader charge analysis or differential charge density plots), as well as the charge transfer mechanism chaotization techniques, e.g., via photoluminescence or transient absorption spectroscopy, are considered as a future extension to this work.

### Photochemical and electrochemical results

Photocurrent measurements were conducted to evaluate the photochemical performance of the synthesized materials under simulated solar illumination. Photocurrent refers to the current generated by the photoelectrode upon light exposure and serves as a key indicator of the material’s efficiency in converting light energy into electrical charge to drive chemical reactions, such as water splitting. To ensure consistency and comparability, photocurrent was expressed as current density (µA/cm²), normalized to the electrode’s surface area (1 × 1 cm^2^). Open Circuit Potential (OCP) analysis was first employed under light ON/OFF cycling without applying any external bias. This method allowed us to monitor voltage fluctuations in response to illumination, providing insights into the charge separation and recombination behavior of the materials. Subsequently, CA was performed at a fixed potential of 0 V vs. RHE using a solar simulator (ABET ANS) under alternating dark and light conditions to mimic real solar irradiation. This experiment involved periodic light-on/off cycles (every ~ 200 s) to record the transient photocurrent response of each material.

**Fig. 11:**
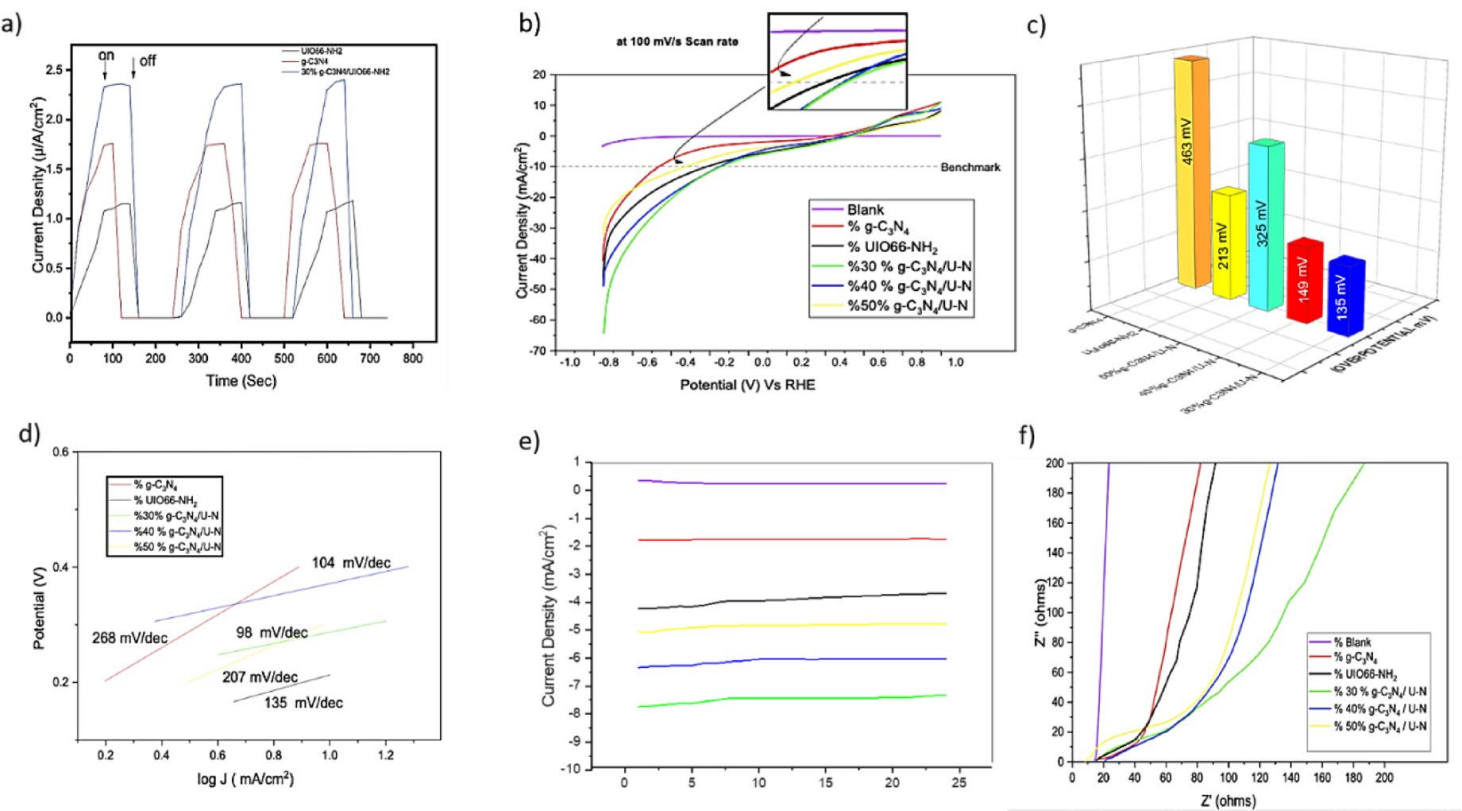
(a) Transient photocurrent response and (CA) was conducted at a fixed potential of 0 V vs. RHE in 0.5 M sodium sulfite (Na₂SO₃) electrolyte at pH 10, using a solar simulator (ABET ANS). The transient photocurrent measurements were recorded under alternating light and dark conditions (~ 200 cycles). (b)(LSV) curves of prepared electrodes deposited on FTO glass for the HER in 1 M KOH (pH 14) at a scan rate of 100 mV/s. The tested electrodes include pure NH₂-UiO-66, pure g-C₃N₄, and three g-C₃N₄/NH₂-UiO-66 composite materials: 30 wt% g-C₃N₄/U-N, 40 wt% g-C₃N₄/U-N, and 50 wt% g-C₃N₄/U-N. All potentials were measured versus the Ag/AgCl (3 M KCl) reference electrode. The overpotential required to reach a current density of 10 mA/cm², a standard benchmark for evaluating HER activity, is highlighted to compare the catalytic performance of each electrode. (b) Overpotential values at a current density of 10 mA/cm² for the (HER). (c) Tafel slopes for the (HER), obtained by plotting overpotential (η) versus the logarithm of current density (log J) from LSV. The Tafel slope values extracted from the linear regions reflect the reaction kinetics, where smaller slopes indicate faster HER kinetics. Electrode (d) (CA) curves of the prepared electrodes deposited on FTO glass for the (HER), conducted at a constant applied potential of − 0.2 V vs. Ag/AgCl for 7 h (e) Nyquist plot measured via at − 0.3 V vs. Ag/AgCl. Measurements were conducted with a 10 mV AC amplitude over a frequency range of 100 kHz to 0.1 Hz. To further verify structural stability, post-stability XRD analysis was performed on the best-performing composite, with the results presented in Supplementary Figure S2.

The photocurrent-time ($\:I-t$) response under intermittent Light exposure demonstrates the enhanced photochemical efficacy of the 30% g-C₃N₄/UiO66-NH₂ composite relative to the unmodified materials. As deliberated in Fig. 11-a, upon exposure to light, all samples demonstrate an enhancement in photocurrent, signifying photoactivity; yet, the composite displays the highest current density (2.5 µA/cm²), followed by g-C₃N₄ (1.8 µA/cm²) and UiO66-NH₂ (1.2 µA/cm²). The improved performance is due to the establishment of an effective heterojunction between g-C₃N₄ and UiO66-NH₂, which promotes greater charge separation and transfer, hence diminishing the recombination of photogenerated electron–hole pairs. The optimized 30% loading of g-C₃N₄ in the composite ensures enough light absorption and effective interfacial interaction without blocking the active catalytic sites, as confirmed by UV-Vis characterization analysis that it has the lowest band gap. Reported photocurrent densities for UiO-66/g-C₃N₄ composites typically range between 2 and 3 µA cm⁻², with NH₂-UiO-66/g-C₃N₄ heterojunctions showing values around 2.4 µA cm⁻². These findings confirm that our observed value is within the expected operational range for this class of materials^56,57,58^. Although this photocurrent was enough to drive hydrogen evolution, as evidenced by the visible generation of H₂ bubbles during illumination. The formation of a well-integrated heterojunction between g-C₃N₄ and the MOF enhances charge transfer pathways, reduces charge recombination, and facilitates efficient separation and migration of photogenerated electron–hole pairs, thereby extending their lifetimes and improving reaction kinetics. This structural and electronic synergy also contributes to a high surface area and better exposure of active sites, further enhancing light harvesting. The rapid, stable, and repeatable photocurrent response observed across multiple cycles confirms the improved charge carrier dynamics and photocatalytic efficiency of the composite, highlighting its strong potential for solar-driven water splitting and related photoelectrochemical applications.

Moreover, electrochemical measurements were conducted to evaluate the electrochemical performance of the synthesized materials and to enhance the hydrogen evolution efficiency. In the linear sweep voltammetry (LSV) test in Fig. 11-b, pure g-C₃N₄ and UiO-66-NH₂ each exhibit moderate current densities, with g-C₃N₄ showing better performance. The increase in HER performance with higher UiO-66-NH₂ content suggests that UiO-66-NH₂ plays a key role in facilitating charge transport and exposing more accessible active sites, thereby enhancing the overall electrocatalytic efficiency. This improvement is consistent with SEM observations, where the porous framework and uniform g-C₃N₄ dispersion increase electrochemically active surface area and facilitate reactant diffusion to catalytic sites. A 10 mA/cm² was used as a benchmark because it corresponds to the practical target for solar-to-hydrogen conversion and is widely accepted in the HER research community as a standard point to evaluate catalyst performance and required overpotential. Though the overpotential values at a current density of 10 mA/cm² for the hydrogen evolution reaction (HER) are shown in Fig. 11-c. The composite including 50% g-C₃N₄ and 50% UiO-66-NH₂ exhibits an overpotential of 325 mV; the composite with 40% g-C₃N₄ and 60% UiO-66-NH₂ has an overpotential of 149 mV; the composite containing 30% g-C₃N₄ and 70% UiO-66-NH₂ shows an overpotential of 135 mV; pure g-C₃N₄ exhibits an overpotential of 464 mv and the pure UiO-66-NH₂ presents an overpotential of 213 mV. This demonstrates that the composite with a greater proportion of UiO-66-NH₂ (70%) has a markedly reduced overpotential of 135 mV, which surpasses the performance of the 50:50 and 40:60 composites. The enhanced interface between g-C₃N₄ and UiO-66-NH₂, as evidenced by SEM morphology and preserved crystallinity from XRD patterns, likely introduces defect sites and efficient conductive channels that lower the overpotential required for HER.

The Tafel plot in Fig. 11-d depicts the electrocatalytic efficacy of diverse photocatalyst materials, it an indication of reaction kinetics, demonstrating that reduced values correlate with enhanced catalytic activity. Pristine g-C₃N₄ demonstrates the largest Tafel slope (268 mV/dec), signifying suboptimal electrocatalytic performance, whereas UiO-66-NH₂ indicates moderate performance with a slope of 135 mV/dec. The composites, specifically the 30%, 40% and 50% g-C₃N₄/U-N samples, exhibit markedly enhanced performance with Tafel slopes of 98 mV/dec,104 mV/dec, and 207 mV/dec, respectively. This enhancement indicates a robust synergistic interaction between g-C₃N₄ and UiO-66-NH₂, presumably augmenting charge separation and amplifying active sites. Such behavior is supported by the broader light absorption in UV–Vis spectra, which ensures greater photogenerated charge availability for electrochemical reactions, thereby complementing the improved electron transfer pathways. Nevertheless, the 50% g-C₃N₄/U-N composite has an elevated Tafel slope of 207 mV/dec, suggesting that an excess of g-C₃N₄ may hinder charge transfer or diminish catalytic surface area. The results underscore the necessity of optimizing the g-C₃N₄ content in the composite to attain peak catalytic efficiency, with the 30% composition exhibiting the most advantageous reaction kinetics. The ~ 100 mV/dec values for the optimal composites suggest that the HER proceeds mainly via the Volmer–Heyrovsky mechanism, with the electrochemical desorption (Heyrovsky) step being rate-limiting.

Chronoamperometry (Stability) test results shown in Fig. 11-e with time provide the subsequent insights: All tests exhibit relative stability over time. The positive current observed for the blank sample is due to background capacitive charging and possibly minor oxidation of residual species in the electrolyte. Since the FTO alone lacks any catalytic activity for HER, there is no significant hydrogen generation, and the current remains in the positive (oxidation) region. This behavior confirms that the catalytic current observed in the active samples arises from the catalyst and not from the substrate or electrolyte itself. Moreover, the composite consisting of 30% g-C₃N₄ and 70% UiO-66-NH₂ exhibits the highest current density over the 7-hour testing period; in contrast, the pure g-C₃N₄ and UiO-66-NH₂ show the lowest current densities. Furthermore, composites with intermediate ratios (30%, 40%, and 50% g-C₃N₄) reveal current densities that are intermediate between those of the pure components and the 70:30 composite. Moreover, we have conducted BET analysis and included the N₂ adsorption-desorption isotherms in Supplementary Materials, see Figure S3. The BET surface area of the 70:30 composite was found to be 172,965 m² g⁻¹, which supports the high catalytic performance by providing more active sites. The mass of active material per unit area was quantified (0.42 mg cm⁻²), and the current densities were recalculated accordingly, see Figure S4 in the Supplementary material.

7 $$\:Degradation\:Rate\:\left(\text{\%}\right)=\left(\frac{J\:inital-J\:final}{J\:inital\:}\right)\times\:100$$

To evaluate the long-term electrocatalytic stability of the synthesized materials, current density degradation rates were calculated over the test period. The blank sample exhibited a notable degradation rate of approximately 35.9%, reflecting its limited catalytic contribution. Among the pure materials, g-C₃N₄ showed the highest stability with a degradation rate of only 6.0%, while UiO-66-NH₂ degraded by 28.0%, Likely due to its lower intrinsic conductivity. In the composite samples, the 50% g-C₃N₄/UiO-66-NH₂ composite demonstrated superior stability with a degradation rate of 7.5%, followed by the 30% g-C₃N₄/UiO-66-NH₂ composite at 10.8%. The 40% composite, by comparison, showed a higher degradation of 17.0%, suggesting that optimized ratios of g-C₃N₄ enhance both activity and durability of the hybrid catalyst system. Alternatively, Electrochemical Impedance Spectroscopy (EIS) is a powerful and widely used technique for analyzing interfacial properties and charge transfer processes at the electrode/electrolyte interface. EIS is particularly valuable in studying electrocatalytic reactions such as HER and the oxygen evolution reaction (OER). EIS measures the impedance (resistance to current flow) of an electrochemical system over a range of alternating current (AC) frequencies. EIS was performed at a frequency range from 100 kHz to 0.1 Hz with a 10 mV AC amplitude and at − 0.3 V vs. Ag/AgCl to ensure that the measurement captured the onset region of HER activity.

To calculate the equivalent circuit fitting and quantitative resistance values from the (EIS) test, the data obtained over the frequency range of 100 kHz to 0.1 Hz, with a 10 mV AC amplitude at − 0.3 V vs. Ag/AgCl, was analyzed. The Nyquist plot generated from the EIS data typically reveals a semicircle at higher frequencies, corresponding to charge transfer resistance$\:{R}_{ct}$, and a linear region at lower frequencies, indicative of Warburg impedance$\:{Z}_{w}$). By fitting the experimental data to an appropriate equivalent circuit model, we extracted the following resistance values: the charge transfer resistance ($\:{R}_{ct}$) was found to be approximately 45 Ω, indicating the kinetic barrier for the hydrogen evolution reaction at the electrode interface; the solution resistance ($\:{R}_{s}$) was determined to be around 8 Ω; and the Warburg impedance coefficient (σ) was measured as 0.25 Ω s^0.5, providing insights into mass transport Limitations. Overall, these values agree with the Literature shown in e 1. The proposed potential balance catalytic relevance with signal stability and avoids interference from excessive gas evolution. On the other hand, Nyquist plots demonstrated that the 70:30 composite had the smallest semicircle diameter, reflecting the lowest charge transfer resistance and more efficient electron transport, as shown in Fig. 11-f. This reduced$\:{\:(R}_{ct}$). aligns with the structural integrity confirmed by XRD and the SEM-indicated interconnected framework, which together maintain conductive pathways and minimize energy barriers for electron transfer. To ensure reproducibility, all photochemical and electrochemical measurements were repeated three times under identical conditions, and the standard deviation was found to be within ± 5% across replicates.

The superior performance of the composite material compared to the pure components can be attributed to several synergistic factors. First, the reduced charge transfer resistance facilitates faster electron transport across the interface, which enhances the overall electrochemical kinetics. Additionally, the improved electrical conductivity of the composite supports efficient charge mobility throughout the catalyst matrix. The optimized exposure of catalytic active sites ensures greater accessibility for reactants, thereby increasing the reaction rate. Moreover, the composite structure promotes enhanced H⁺ adsorption and activation, which is crucial for accelerating the hydrogen evolution reaction and improving the catalytic efficiency under electrochemical conditions. When these electrochemical results are viewed alongside the structural and optical characterizations, a clear correlation emerges that the combined morphological features, crystallinity retention, and broadened light absorption are directly responsible for the observed HER improvements. A comparative analysis of various HER electrocatalysts under different electrolyte conditions shown in Table 1, reveals notable differences in performance metrics such as overpotential and Tafel slope. Among the surveyed materials, the 30% g-C₃N₄/UiO-66-NH₂ (U-N) composite developed in this work exhibited superior catalytic activity in alkaline media, achieving a low overpotential of 135 mV at − 10 mA/cm² and a favorable Tafel slope of ~ 98 mV/dec. These values surpass those of previously reported UIO-based systems and other carbon nitride composites, indicating enhanced HER kinetics and improved interfacial charge transfer. This performance underscores the effectiveness of our rational composite design strategy and positions the synthesized material as a promising non-precious HER catalyst.

**Table 1:** Comparative study for electrochemical measurements for HER.

Catalyst	Electrolyte	Performance Metrics	Ref
Zr-MOF/BiVO₄ Nanocomposite	1.0 M KOH electrolyte solution.	Overpotential (HER): 300 mV at 10 mA/cm²; Band gap: 3.56 eV.	^57^
c-Ru@H-NPC (Ru on N-doped hollow carbon)	1 M KOH & alkaline seawater	Overpotential (HER): 10 mV (alkaline)/12 mV (alkaline seawater) at 10 mA/cm²;	^58^
P-doped g-C₃N₄/MoS₂	Acidic	Tafel slopes (mV/dec): - P20%-NV-g-C₃N₄: 144- MoS₂: 101.2- g-C₃N₄/MoS₂: 84.8 - Pt/C: 51.1- P20%-NV-g-C₃N₄/MoS₂: 70.7 (lowest among non-Pt)	^59^
CoMn-LDH@g-C₃N₄ Nanohybrids	Alkaline (1.0 M KOH)	Overpotential (η₁₀): - CoMn-LDH@g-C₃N₄: 406 mV- CoMn-LDH: 491 mV- g-C₃N₄: 670 Mv	^60^
Ultrafine g-C₃N₄ Quantum Dots (QDs)	Acidic (0.5 M H₂SO₄)	Overpotential at 10 mA/cm²: ~208 mV	^61^
NH₂-UiO-66/CuO Nanocomposite	Alkaline (1.0 M KOH)	Overpotential (η₁₀): 176 mV Tafel slope: 87 mV/dec Measured via CV (- −0.4 to 1.0 V vs. RHE)	^62^
Nano UiO-66 vs. UiO-66-NH₂ (on NF substrate)	Alkaline (1.0 M KOH)	Overpotential at −10 mA/cm²: - UiO-66 (U): 143 mV - UiO-66-NH₂ (U-N): 179 mV Tafel slope: - U: 117 mV/dec - U-N: 218 mV/dec	^28^
30% gC3N4/U-N	Alkaline (1.0 M KOH)	- over potential (135 mV) at −10 mA/cm² -HER kinetics (Tafel slope ~ 98 mV/dec)	**This work**

## Conclusion

The advancement of innovative materials and catalytic systems has become paramount in enhancing the performance and efficiency of water-splitting technologies, striving to meet the escalating energy demands while minimizing environmental impact. Although previous studies have established the potential of metal-organic frameworks (MOFs)—particularly UiO-66-NH₂—as promising catalysts for the hydrogen evolution reaction (HER), a comprehensive understanding of the mechanisms governing their performance remains elusive. In many instances, it is unclear whether the enhanced catalytic activity observed in MOF-based composites stems from the intrinsic properties of the MOF itself or from synergistic interactions within the heterostructure. This ambiguity highlights the necessity for integrative studies that amalgamate experimental measurements with theoretical modeling to elucidate the fundamental principles underlying improved performance. This work addresses this critical gap by systematically investigating both the electrochemical behavior and the electronic structure of UiO-66-NH₂/g-C₃N₄ composites. The integration of experimental findings with density functional theory (DFT) simulations provides a comprehensive understanding of how structural, electronic, and interfacial factors contribute to HER performance.

This study demonstrates the successful synthesis and thorough evaluation of UiO-66-NH₂/g-C₃N₄ composite materials for HER under both electrochemical and photoelectrochemical conditions. Among the tested ratios, the composite comprising 70 wt% UiO-66-NH₂ and 30 wt% g-C₃N₄ exhibited the most promising HER activity, showcasing a low overpotential of approximately 135 mV at a current density of 10 mA/cm², thereby outperforming the 60:40 and 50:50 composites, which required higher overpotentials of ~ 194 mV and ~ 213 mV, respectively. Tafel slope analysis revealed improved HER kinetics for the 70:30 composite, with a lower slope of ~ 98 mV/dec compared to 104 mV/dec and 207 mV/dec for the 60:40 and 50:50 ratios, respectively. Furthermore, photochemical analyses consistently demonstrated that the composite containing 70 wt% UiO-66-NH₂ and 30 wt% g-C₃N₄ exhibited the highest and most stable photocurrent, affirming its superior photo and electrocatalytic activity relative to the other compositions. To ensure reproducibility, all photochemical and electrochemical measurements were repeated three times under identical conditions, and the standard deviation was found to be within ± 5% across replicates. Moreover, the composite displayed pronounced photoelectrochemical behavior that aligned seamlessly with its bandgap characteristics, as corroborated by DFT simulations. This composite synergistically combines the advantages of both materials: the visible-light responsiveness of g-C₃N₄ and the enhanced charge transport capabilities of UiO-66-NH₂, with aligned band edges facilitating efficient electron-hole separation and minimizing recombination. Ultimately, the theoretical insights obtained corroborated the experimental data, confirming the effective fabrication of the catalyst. Consequently, this study not only presents an effective photocatalytic material but also contributes valuable insights into design strategies for future MOF-based hybrid systems in renewable energy applications.

## Supplementary Information

Below is the link to the electronic supplementary material.

Supplementary Material 1

## Data Availability

The datasets generated and analyzed during the current study are available from the corresponding author upon reasonable request.

## Code availability

Not applicable for that section.
