# Supplementary material for: Photochemical and electrochemical assessment of UIO-66-NH₂/g-C₃N₄ thin-film heterostructures as potential candidates for hydrogen evolution: an experimental study augmented by DFT insights
Source: Sci Rep. 2025 Sep 18;15:32608. doi: 10.1038/s41598-025-20035-4 (PMC12446450; doi:10.1038/s41598-025-20035-4)
Supplement: Supplementary file 1 — Supplementary Material 1 [file 41598_2025_20035_MOESM1_ESM.pdf]

Photochemical and Electrochemical Assessment of UIO-66-NH_2/g-C_3N_4 Thin-Film Heterostructures as Potential Candidates for Hydrogen Evolution: An Experimental Study Augmented by DFT Insights

Nour AbouSeada[1], Maryam G. Elmahgary[2], Sameh O. Abdellatif[3]*, and Khaled Kirah[4].
[1] The Faculty of Energy and Environmental Engineering, the British University in Egypt (BUE), El-Sherouk City, Cairo 11837, Egypt.
[2] The Chemical Engineering department, British University in Egypt (BUE), 11387, Cairo, Egypt.
[3] The Electrical Engineering department, and FabLab, at the Centre of Emerging Learning Technologies, CELT, British University in Egypt (BUE), 11387, Cairo, Egypt.
[4] Engineering Physics Department, Faculty of Engineering, Ain Shams University, Cairo, Egypt.
*Corresponding author: Sameh O. Abdellatif (e-mail: sameh.osama@bue.edu.eg).

Supplementary material

S1. Mott–Schottky Analysis

From the Mott–Schottky analysis, the flat-band potentials were –1.00 V for g-C_3N_4, –0.65 V for UiO-66-NH_2, and –0.80 V for the 30 % g-C_3N_4/UiO-66-NH_2 composite (vs. Ag/AgCl). Converting to the NHE scale and combining with the respective band gaps (g-C_3N_4 ≈ 2.75 eV, UiO-66-NH_2 ≈ 2.83 eV, and the composite ≈ 2.63 eV), the conduction and valence band positions confirm a staggered arrangement, where the CB of g-C_3N_4 is more negative and the VB of UiO-66-NH_2 is more positive. This staggered alignment enables spatial separation of electrons and holes, which is characteristic of a Type-II heterojunction, thus validating our heterostructure band configuration.

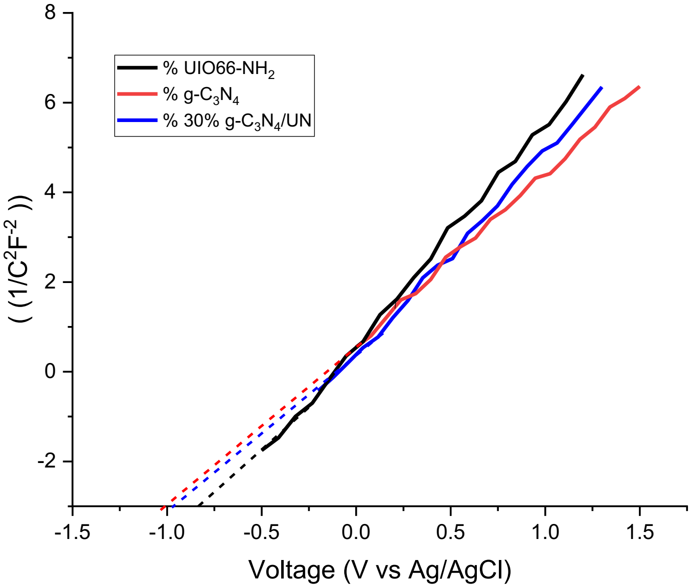

Figure S2 Mott–Schottky (M–S) plots of UiO-66-NH_2, g-C_3N_4, and the 30% g-C_3N_4/UiO-66-NH_2 composite measured in 0.5 M Na_2SO_4 electrolyte (pH ≈ 7) at a frequency of 1 kHz (vs. Ag/AgCl). The intercepts of the extrapolated linear regions were used to determine the f flat-band potentials, which were subsequently converted to the NHE scale for band alignment analysis.

S2. The XRD Analysis

The structural stability of the 30% g-C_3N_4/U-N composite was evaluated by comparing its X-ray diffraction (XRD) patterns before and after a 24 h stability test. As shown in Figure S2, the diffraction peaks corresponding to the characteristic reflections of UiO-66-NH_2 (e.g., at $2\theta \approx 7.4°$) and g-C_3N_4 (broad feature at $2\theta \approx 27.5°$) are clearly observed in both patterns. The nearly identical peak positions and intensities before and after the stability test indicate that the composite maintained its crystallographic structure without noticeable lattice distortion or loss of crystallinity. Only minimal differences in peak broadening or intensity were observed, which can be attributed to minor surface modifications rather than structural degradation

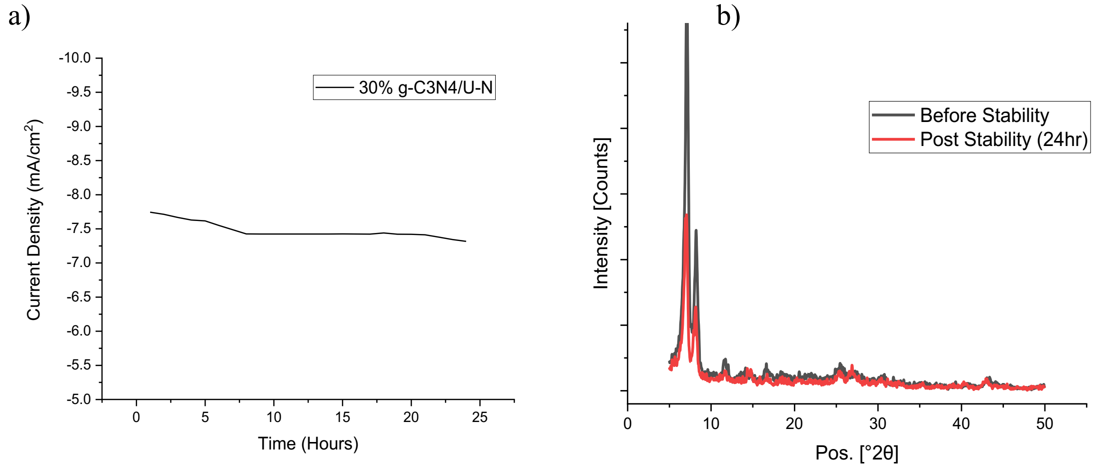

Figure S2 a) (CA) curves of the campsite deposited on FTO glass for the (HER), conducted at a constant applied potential of −0.2 V vs. Ag/AgCl for 24 hours b) XRD patterns of the optimal g-C_3N_4/UiO-66-NH_2 composite before and after 24 h chronoamperometric stability testing, showing no significant changes in peak positions or intensities, indicating good structural stability of the catalyst.

S3. The Brunauer–Emmett–Teller (BET) Analysis

The nitrogen adsorption–desorption isotherms of the MOF/g-C_3N_4 composite were analyzed using the Brunauer–Emmett–Teller (BET) model within the relative pressure range of 0.05–0.30. The BET plot exhibited a strong linear correlation ($R^2 = 0.98812$), confirming the appropriateness of the model for the sample. From the slope (0.02457) and intercept (5.9451×10^{-4}) of the linear fit, the monolayer adsorbed gas quantity was calculated to be 39.74 $cm^3\ g^{-1}$, while the BET constant (CCC) was determined as 42.33, indicating strong interactions between nitrogen molecules and the adsorbent surface. The specific surface area was calculated as 1729.65 $m^2\ g^{-1}$, revealing the exceptionally high porosity of the composite, which is advantageous for enhancing active site accessibility and mass transport in photocatalytic applications.

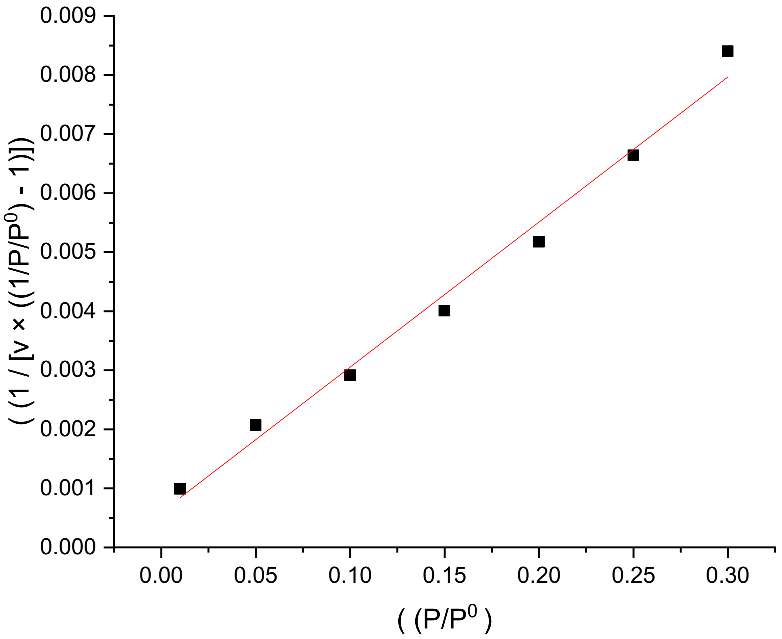

Figure S3 The nitrogen adsorption–desorption data of the MOF/g-C_3N_4 composite

S4. Mass-normalized linear sweep voltammetry Analysis

The recalculated values, based on the quantified active mass loading of 0.42 mg cm^{-2}, enable an accurate comparison of catalytic activities across different systems and loadings by removing the geometric bias.

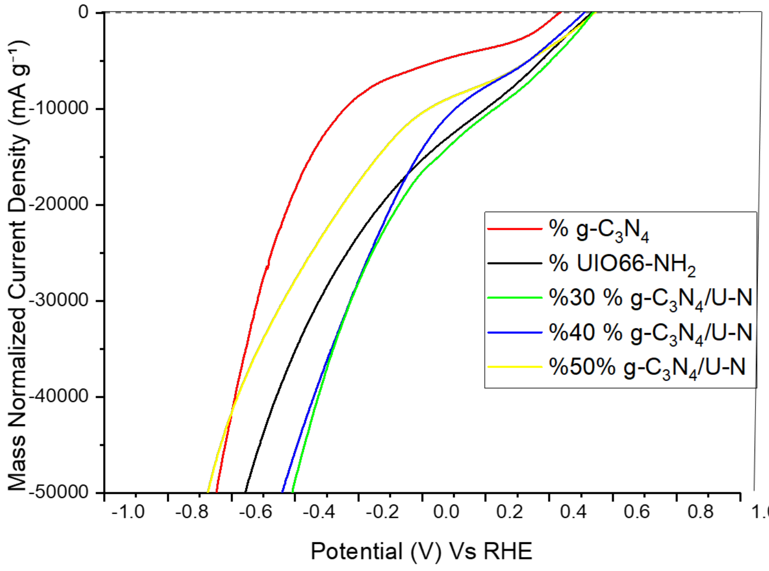

Figure S3 Mass-normalized linear sweep voltammetry (LSV) curves of UiO-66-NH_2, g-C_3N_4, and g-C_3N_4/UiO-66-NH_2 composites.
